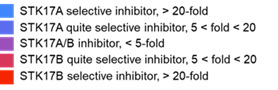# STK17A (DRAK1) at the crossroads of apoptosis, immunity, and cancer: Emerging roles and therapeutic opportunities

**DOI:** 10.7150/thno.129910

**Published:** 2026-03-30

**Authors:** Leticia Christina Pires Gonçalves, Olivia Rastoin, Vira Morozova, Clément Buzet, Audrey Bennetot, Gilles Pagès, Cyril Ronco, Maeva Dufies

**Affiliations:** 1 Roca Therapeutics, Nice, France.; 2 University Côte d'Azur, Institut de Chimie de Nice (ICN), CNRS UMR 7272, Nice, France.; 3 V. N. Karazin Kharkiv National University, School of Chemistry, Kharkiv, Ukraine.; 4 University Côte d'Azur, Institute for Research on Cancer and Aging of Nice (IRCAN), CNRS UMR 7284, INSERM U1081, Nice, France.; 5 Institut Universitaire de France (IUF), Paris, France.

## Abstract

Death-associated protein kinase-Related Apoptosis-inducing protein Kinase 1 (DRAK1/STK17A) is a serine/threonine kinase of the Death Associated Protein Kinase (DAPK) family. STK17A is widely expressed and enriched in immune tissues, and is primarily localized in the nucleus, though it can translocate to the cytoplasm in response to specific stimuli. STK17A stimulates apoptosis and cytoskeletal dynamics, but its physiological roles remain incompletely defined, in part due to limited availability of potent/selective chemical probes and the absence of STK17A in commonly used rodent models.

In this review, we summarize current knowledge on STK17A, including its structure, evolution, expression patterns, molecular interactions, and roles in cancer as well as in autoimmune, cardiovascular, infectious, and neurological disorders. We also compare STK17A with its closest homolog, STK17B, highlighting both shared features and functional distinctions.

The review further examines recent medicinal chemistry efforts that have yielded the first small-molecule modulators of STK17A (DRAK1) and STK17B (DRAK2), including dual inhibitors and emerging selective scaffolds. These compounds can serve as valuable chemical probes and hold promising therapeutic potential. Nonetheless, challenges of selectivity and functional validation remain, emphasizing the need for continued medicinal chemistry efforts to unlock the full potential of STK17A as a therapeutic target across cancer, autoimmune, and neurodegenerative diseases.

## Introduction

Protein kinases play a fundamental role in regulating cellular signaling and metabolic pathways. Their activity is essential for processes such as cell proliferation, differentiation, and apoptosis. Dysregulation of kinase function has been implicated in a wide range of diseases, including cancer, inflammatory conditions and neurodegenerative disorders. Understanding kinase structure and function, therefore, remains a central focus in biomedical research, and modulation of their activity offers significant therapeutic potential for addressing diverse pathologies. Within the broad family of kinases, the calcium/calmodulin-dependent protein kinases (CAMKs), and particularly their subfamilies, occupy a central role in apoptosis and cytoskeletal regulation. The CAMKs are composed of three main subfamilies: the Death-Associated Protein Kinases (DAPKs), the Myosin Light chain-related Kinases (MLKs), and the TRIple functional dOmain protein-related kinases (TRIOs), all of which are implicated in apoptotic processes, cytoskeletal dynamics, and cellular plasticity. Although classified within the CAMK superfamily, only about half of the kinases in the subfamilies possess an additional C-terminal autoinhibitory domain capable of binding calcium/calmodulin to regulate kinase activity. Instead, many are regulated through alternative mechanisms, including phosphorylation or interaction with other protein ligands 1.

DAPKs are a subset of calcium/calmodulin (CaM)-dependent serine/threonine kinases localized in the cytoskeleton, characterized by the presence of a death domain, and promote apoptotic signaling. This family includes five kinases: DAPK1, DAPK2, DAPK3 (also known as ZIPK), and the less-characterized STK17A (DRAK1) and STK17B (DRAK2). Importantly, overexpression of DAPK family members induces direct cell death in multiple cell lines in a kinase activity-dependent manner 2. Their kinase domain is situated near the N-terminus, and DAPK1, DAPK2, and DAPK3 share sequence motifs within the upper lobe of their catalytic domains. DAPK1 and DAPK2 are more closely related to DAPK3 and possess a calcium/calmodulin-binding autoinhibitory domain, unlike DAPK3, which instead contains three serine/threonine phosphorylation sites within its catalytic domain 1.

The DAPK family contributes to apoptosis notably via tumor necrosis factor-alpha (TNF-α) and Fas-induced cell death pathways 3. Additionally, DAPK activity impairs cell adhesion by inhibiting integrin functions and survival signaling pathways, thereby activating p53. Integrins mediate the connection between the extracellular matrix and the cytoskeleton and promote survival pathways such as focal adhesion kinase (FAK) activation 4. DAPK activity also exerts anti-metastatic effects by impeding the survival of anchorage-dependent cells 5.

The catalytic domain homology and regulatory mechanisms of DAPK family members are sufficiently conserved to classify them as a distinct serine/threonine kinase subfamily. Within this group, STK17A, STK17B and DAPK3, lack the CaM domain and are predominantly localized in the nucleus 6. Although several functions have been described, this protein family remains poorly characterized and likely carries out additional roles that are not yet fully understood.

Within this context, STK17A emerges as one of the least studied members of the DAPK family.

Unlike other members of the death-associated protein kinase family, STK17A exhibits several distinctive features that complicate its biological and translational interpretation. STK17A lacks the death domain and calcium/calmodulin regulatory motifs characteristic of DAPK1-3, and its gene is absent in murid genomes, precluding conventional genetic interrogation in widely used mouse models. As a result, much of the current literature relies on overexpression or knockdown approaches in transformed cell lines, raising important questions regarding physiological relevance. Additional bottlenecks include limited validation of endogenous kinase activity and substrates, frequent reliance on transcriptomic or network-based associations, and apparent context-dependent functional outputs that vary across cellular and disease settings. Together, these features distinguish STK17A from other death-associated kinases and underscore the need for cautious interpretation of existing data, as well as for improved experimental and chemical tools to resolve its precise biological roles.

Accordingly, this review focuses on STK17A, with brief comparisons to STK17B, describing their structure and distribution, summarizing their molecular interactions, and highlighting their roles in cancer and other human diseases. In addition, we provide a comprehensive and critical analysis of all known or designed small molecules that modulate their activity.

## The Structure of STK17A and STK17B

Death-associated protein kinase-Related Apoptosis-inducing protein Kinase 1 and 2 (DRAK1/STK17A and DRAK2/STK17B, respectively) are two serine/threonine kinases belonging to the DAPK family, first discovered in 1998 7. Also known respectively as serine/threonine kinase 17A (STK17A) and serine/threonine kinase 17B (STK17B), they are ubiquitously expressed across tissues.

Structurally, STK17A and STK17B possess catalytic domains that are highly homologous to those of DAPK1 and DAPK3. However, they lack several regulatory domains present in DAPK1, notably the death domain and the calcium/calmodulin-binding regulatory domain, which are critical for canonical DAPK1 regulation 6.

STK17A is encoded by the STK17A gene, located on the chromosome 7p13,spanning 44,622 base pairs and comprising seven exons and seven introns 8. STK17B is encoded by the STK17B gene, located on the chromosome 2q32.3. The corresponding open reading frames encode a protein of 414 amino acids for STK17A and 372 amino acids for STK17B. Both kinases contain N-terminal kinase domains, sharing approximately 67% sequence homology, and encompassing 11 conserved subdomains typical of serine/threonine kinases which are found across the DAPK family, as well as other CAMK-related kinases such as CaMKII and MLCK.

At the sequence level, the catalytic domains of STK17A and STK17B harbor all canonical motifs required for kinase activity, although their absolute positions differ due to variations in N-terminal length. In STK17A, the glycine-rich loop (subdomain I) is defined by the motif GRGKFA at amino acids 68-73, marking the functional onset of the kinase domain, whereas the equivalent motif in STK17B is located at amino acids 40-45. The catalytic lysine within subdomain II is positioned at residues 80-81 in STK17A and 65-66 in STK17B, enabling ATP coordination. The conserved catalytic loop motif HLD (subdomain VI) is present at residues 184-186 in STK17A and 156-158 in STK17B, followed by the activation loop DFG motif (subdomain VII) at residues 207-209 and 179-181, respectively. The activation segment terminates with the conserved APE motif (subdomain VIII) at residues 231-233 in STK17A and 203-205 in STK17B, collectively defining a single, conserved kinase fold in both proteins.

Although STK17A contains an additional glycine-rich sequence (GSGRAG, residues 19-24) upstream of its catalytic domain, this motif is not embedded within a complete kinase fold and lacks accompanying catalytic elements and therefore does not constitute a second functional ATP-binding kinase domain.

**Figure 1:**
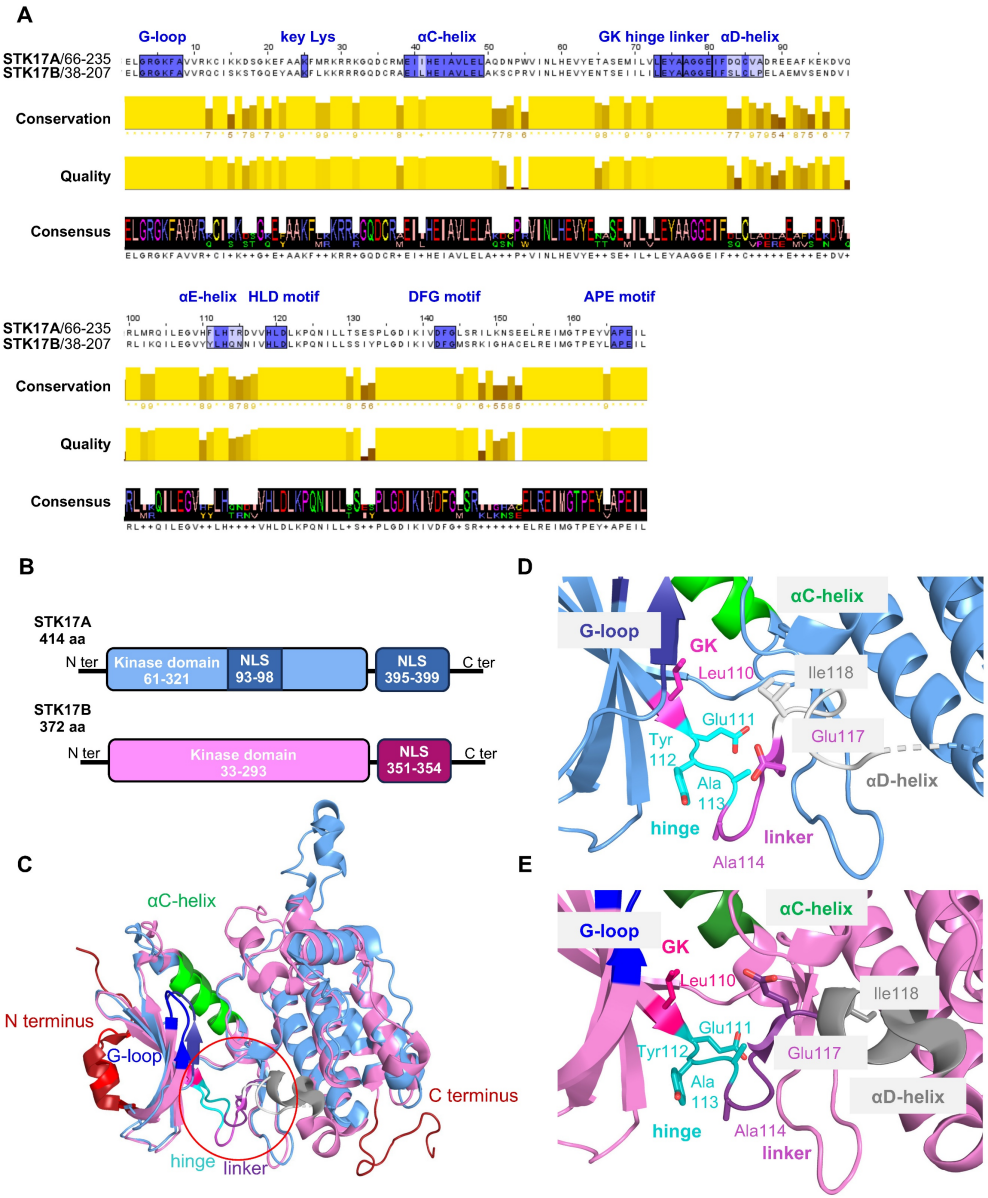
** Structure of STK17A and STK17B proteins.** (A) Amino acids sequence alignment of STK17A and STK17B. MUSCLE algorithm was used to align the full sequences of STK17A (1-414) and STK17B (1-372), followed by pairwise alignment to determine the percentage of identity between STK17A and STK17B. The important residues for the catalytic activity are colored in blue. (B) Schematic representation of the kinase and NLS (nuclear localization signals) domains. (C) 3D structure superimposition of STK17A (blue - PDB 7QUF) and STK17B (magenta - PDB 6ZJF). The catalytic pocket is represented by a red circle. (D) and (E) 3D structure of the structural divergent region and key amino acids of STK17A (blue, D) and STK17B (magenta, E).

Outside the catalytic domain, STK17A and STK17B share ~20-24% sequence homology within their conserved C-terminal region outside the kinase domain (C-terminal tail region; see full-length alignment in **Figure 1A**) 9. This region is comparatively divergent and is not shared with other serine/threonine kinases, suggesting potential roles in localization and protein-protein interactions.

A notable feature of STK17A is the presence of two predicted nuclear localization signals (NLS): one proximal to the kinase domain (amino acids 93-97, sequence RKRRK) and a second within the C-terminal region (amino acids 396-399, sequence KRFK). In contrast, STK17B contains a single C-terminal NLS (amino acids 351-354, sequence KRFR). The presence of these NLS motifs enables predominant nuclear localization of both kinases, although protein kinase C (PKC) activation has been reported to promote their context-dependent translocation to the cytoplasm 1,7,10.

The ATP-binding sites of STK17A and STK17B are highly conserved (**Figure 1A**), not only between the two kinases but also across the broader DAPK family 11. Nevertheless, sequence alignment reveals notable differences in the αD-helix, αE-helix, and xDGF+1 subdomains, with the αD-helix showing the most pronounced divergence (**Figure 1A**). Functionally, this helix is critical for stabilizing the catalytic pocket and orienting residues that interact with ATP. These structural differences in STK17A and STK17B result in substantial changes in the architecture and volume of the catalytic pocket (∼349 Å for STK17A vs. ∼708 Å for STK17) and appear to influence the conformations of the linker and hinge regions, albeit to a lesser extent (**Figure 1D-E**). Despite these differences, the residues directly involved in catalysis are conserved, underscoring the αD-helix as the primary structural divergence between the two kinases. These distinct structural features may be exploited for the rational design of selective inhibitors targeting STK17A or STK17B, by specifically interacting with residues of the αD-helix.

## STK17 and Its Evolution in Eukaryotes

STK17A is absent in two of the most commonly used animal research models, the mouse (*Mus musculus*) and the rat (*Rattus norvegicus*) 12, which severely limits the availability of genetic *in vivo* validation. Although STK17B is present in these species, there is currently no direct experimental evidence demonstrating functional compensation for STK17A loss. It has been proposed that the STK17A gene was lost in the evolutionary lineage of murids (rats and mice), a phenomenon known as gene loss or pseudogenization, which is relatively common in evolution. This loss may result from genomic rearrangements involving a synteny break: a 50.4 kb genomic deletion between two chromosomal blocks that are conserved in other species, such as chimpanzees, pigs, and cows 13.

Despite its absence in mice and rat, STK17A is widely conserved across more than 600 animal species (UniProt database). It is present in numerous invertebrate species as well as key vertebrate models, including zebrafish (*Danio rerio*). In zebrafish, STK17A is located on chromosome 24 and contains seven exons, mirroring the human gene structure 14. To date, however, functional studies of STK17A in zebrafish are lacking.

In contrast, STK17A has been studied in several other organisms. In the Japanese flounder (*Paralichthys olivaceus*), STK17A is dominantly expressed in the liver and exhibits pro-apoptotic activity, particularly in response to bacterial infection and inflammatory stimuli 15.

In rabbits, STK17A shares approximately 88% sequence identity with its human ortholog, retains the conserved DAPK catalytic motifs, and localizes to the nucleus. Functionally, it promotes apoptosis when overexpressed in osteoclasts 16. Similar pro-apoptotic roles have been reported in pigs, where STK17A expression is associated with malignant melanoma progression 17, and in chickens, where STK17A polymorphisms correlated with anxiety-like behaviors 18.

In some invertebrates, the DAPK repertoire is reduced to a single family member. For example, in fruit flies (*Drosophila melanogaster*) and silkworms (*Bombyx mori*), only one DAPK family protein, orthologous to STK17, is present 19. In Drosophila, this STK17 ortholog plays a crucial role in cytoskeletal regulation by phosphorylating Spaghetti Squash (Sqh), the fly homolog of the myosin regulatory light chain (MRLC) 20. STK17A-mediated phosphorylation of MRLC is essential for smooth muscle contraction and for regulating cytoskeletal tension 21-23.

Together, evolutionary analyses reveal an unexpectedly high degree of conservation of STK17A's apoptotic and cytoskeletal regulatory functions across diverse species. At the same time, species-specific adaptations gene losses and functional redundancies highlight the evolutionary plasticity of the DAPK family.

## Regulation of STK17A Expression

**Figure 2:**
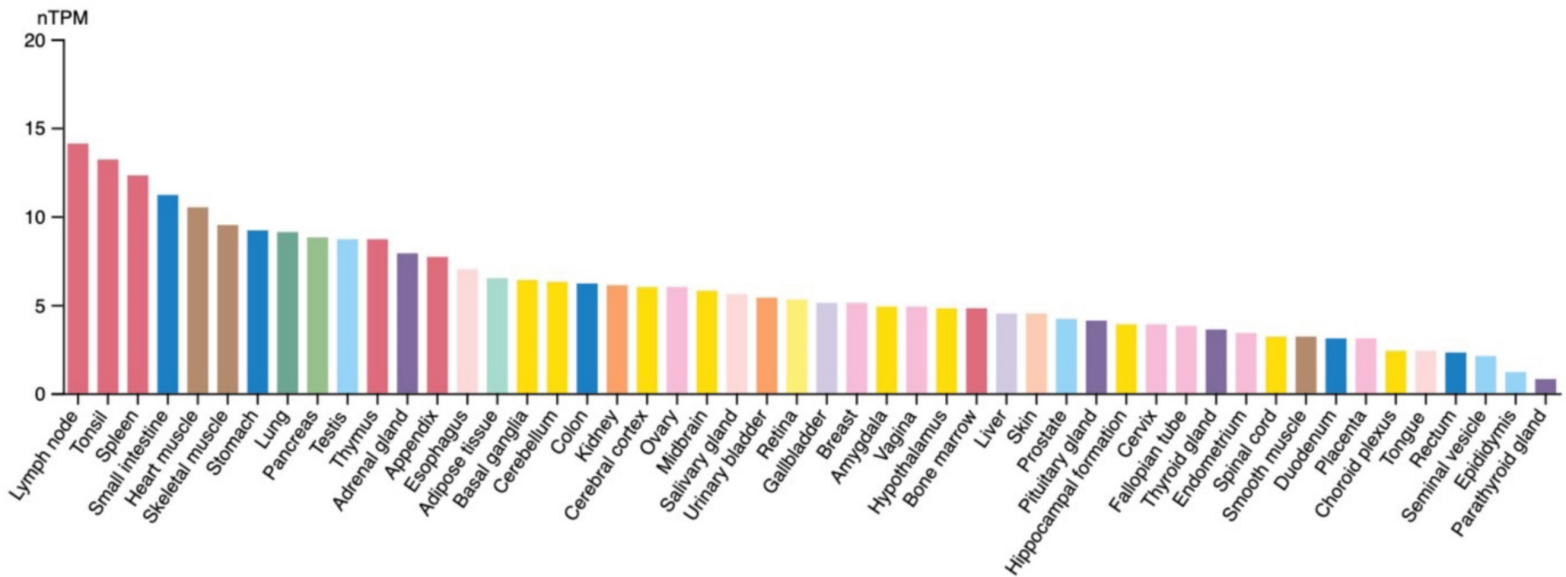
** STK17A mRNA expression in different organs (Protein Atlas**). RNA expression shows RNA-data from two different sources: generated from Human Protein Atlas (HPA) RNA-seq data and RNA seq data from the Genotype-Tissue Expression (GTEx) project. nTPM: relatively comparing RNA level, transcripts per million.

The expression of STK17A is governed by complex regulatory networks involving transcriptional, post-transcriptional, and signaling-dependent mechanisms. Expression profiling (**Figure 2**) indicates that STK17A and STK17B are broadly expressed but show higher transcript abundance in immune-related tissues (e.g., lymph nodes, thymus, spleen).

The expression of STK17A is regulated by multiple upstream signals, including transcription factors, cytokines-triggered pathways, and microRNAs (miRNAs). Throughout this section, we distinguish direct regulation (e.g., promoter binding, response elements) from indirect associations inferred from stress signaling or pharmacologic perturbation.

### Regulation by Transcription Factors

One of the key regulators of STK17A expression is the tumor suppressor p53, a guardian of genomic stability with central roles in DNA damage response, reactive oxygen species (ROS) regulation, senescence, and apoptosis. Upon cellular stress, particularly DNA damage, p53 activates a network of downstream genes that mediate cell-cycle arrest and programmed cell death.

STK17A gene has been identified as a direct transcriptional target of p53, regulated through a p53 response element (p53RE) located near its transcription start site. This direct regulation has been confirmed by promoter analyses and chromatin immunoprecipitation demonstrating p53 binding to the response element 24. p53-dependent upregulation of STK17A has been observed across multiple cell types following exposure to DNA-damaging agents such as cisplatin. This activation may be cell-type dependent, as STK17A does not consistently participate in feedback loop with p53 in testicular cancer cells 25.

This transcriptional axis should be distinguished from reports suggesting that STK17A phosphorylates p53, which—so far—derive from *in vitro* or ectopic-expression. STK17A can phosphorylate p53 at Ser^20^ and Thr^18^. The phosphorylation of p53 is known to stimulate the p53 transcriptional response and plays an important role in controlling its function; thus, making it an interesting target to study 26.

### Regulation by Cytokines

STK17A expression is also induced by cytokine-mediated signaling pathways, notably the type I interferon (IFN-I)/ RNase L pathway. Interferons are critical mediators of antiviral defense and can initiate apoptosis in infected cells. Activation of RNase L down-stream of IFN-I leads to RNA degradation and apoptotic signaling through the c-Jun N-terminal kinase (JNK) pathway.

Studies have shown that IFN-I stimulation, particularly in the presence of double-stranded RNA (dsRNA), results in robust upregulation of STK17A mRNA levels. RNase L-deficient cells exhibit reduced STK17A induction, confirming the importance of this pathway 27. In cell-line models, ectopic STK17A expression has been reported to increase JNK phosphorylation and markers of mitochondrial apoptosis (activation of caspase-3 and cleavage of PARP, and mitochondrial membrane permeabilization through Bax translocation to mitochondria). However, these findings should be interpreted as demonstrating the capacity of STK17A to engage apoptotic signaling under artificial expression conditions, rather than establishing an endogenous requirement.

Collectively, these findings suggested that STK17A overexpression is sufficient to promote intrinsic apoptosis.

### Regulation by microRNAs

At the post-transcriptional level, microRNAs (miRNAs) further modulate STK17A expression. For example, miR-182-5p directly targets STK17A by binding to two distinct sites within its 3' untranslated region (3'UTR), thereby reducing mRNA stability and translation efficiency 12.

Thus, STK17A expression is precisely regulated through an integrated network of transcriptional, cytokine-mediated, and post-transcriptional mechanisms. This multilayered regulation positions STK17A as a central node in the cellular response to stress, DNA damage, and viral infection.

## Molecular Interactions of STK17A

**Figure 3:**
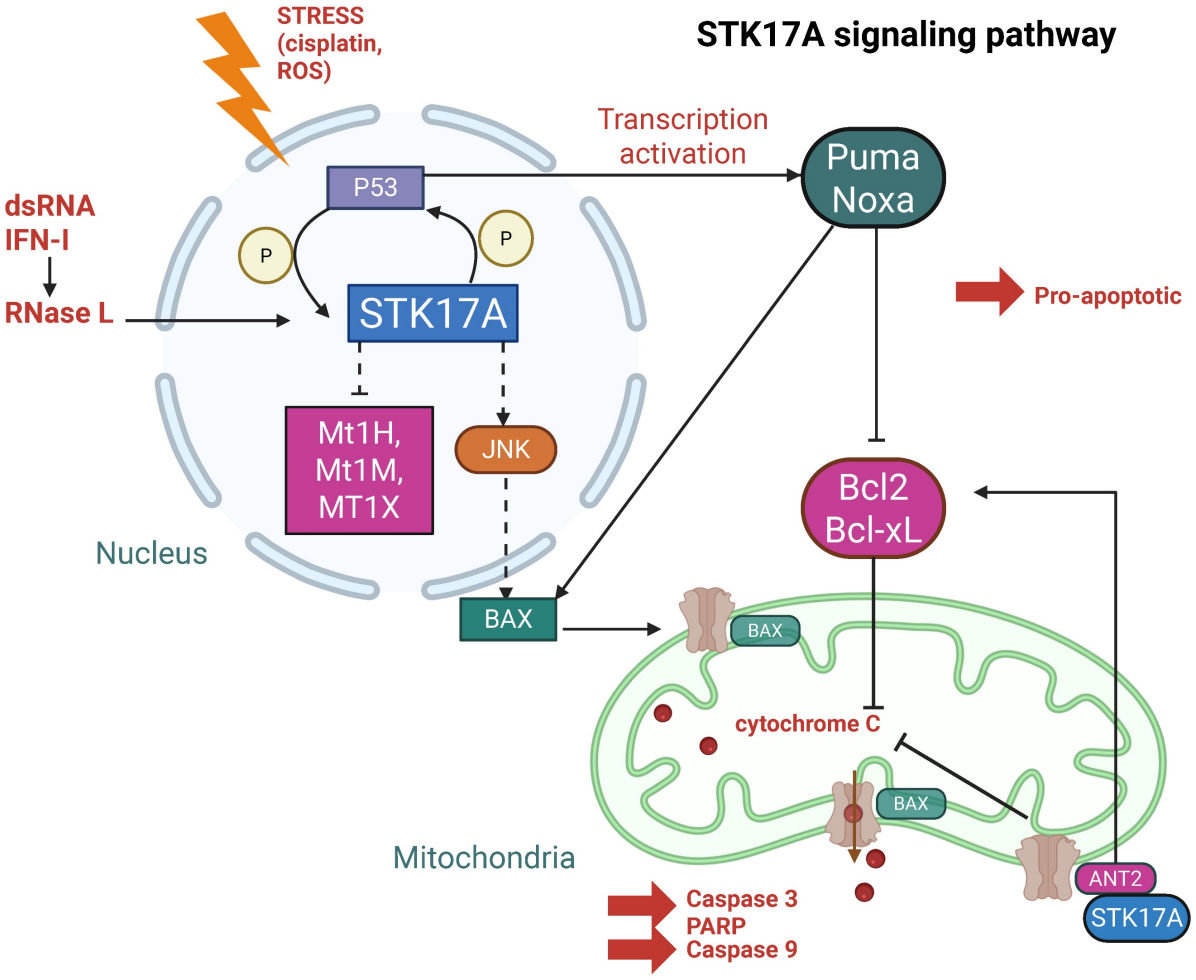
** STK17A and its interactions in the nucleus.** Created with BioRender.com. Solid lines represent reported direct protein-protein interactions, whereas dashed lines indicate indirect regulatory relationships inferred from functional or correlative evidence.

**Figure 4:**
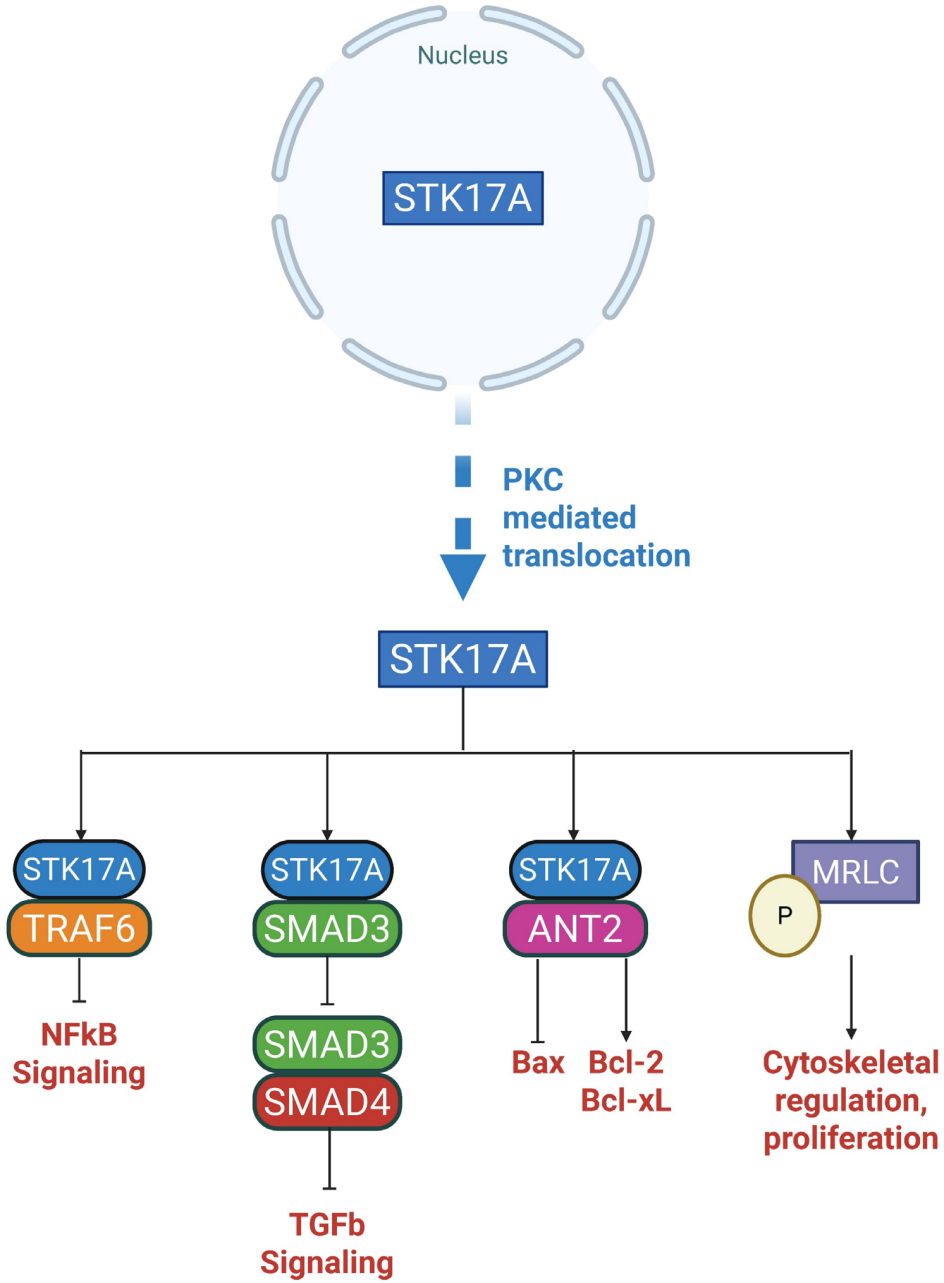
** STK17A and its binding partners in the cytoplasm.** Created with *BioRender.com.* STK17A (STK17A) reported cytoplasmic interactors and putative functional outputs.

Once expressed and/or relocalized, STK17A has been reported to interact with multiple proteins implicated in apoptosis, inflammation, and cytoskeletal regulation (**Figures 3 and 4**). Importantly, most interactions have been identified in overexpression or co-immunoprecipitation settings, and direct kinase-substrate relationships remain largely unvalidated at endogenous levels.

### Nuclear Targets

In the nucleus (**Figure 3**), STK17A exerts its kinase activity, although its endogenous substrates remain largely unidentified. Nuclear STK17A is essential for the activation of apoptotic pathways, particularly through activation of the JNK signaling cascade. Overexpression of STK17A indirectly activates JNK, which subsequently phosphorylates Bax facilitating its translocation. This translocation promotes cytochrome C release and leads to the cleavage of caspase-3 and PARP 27.

Moreover, knockdown of STK17A results in elevated of antioxidation response genes such as Mt1H, Mt1M and Mt1X, suggesting that STK17A contributes to the enhancement of intracellular ROS by repressing these genes 28.

### Cytoplasmic Targets

While predominantly nuclear, STK17A can translocate to the cytoplasm under specific conditions (**Figure 4**), such as activation of Protein Kinase C (PKC). Upon cytoplasmic translocation, STK17A interacts with several important regulatory proteins:

#### Smad3

STK17A binds to Smad3, a key mediator of TGF-β1 signaling. Notably, STK17A binding does not result in Smad3 phosphorylation. Instead, STK17A inhibits the formation of the Smad3/Smad4 complex, thereby blocking the nuclear translocation of Smad3 and the subsequent activation of TGF-β1 target genes. This interference impairs TGF-β1's signaling 29.

#### TRAF6

STK17A also interacts with TNF receptor-associated factor 6 (TRAF6), an E3 ubiquitin ligase critical for the activation of NF-kB signaling and the inflammatory response. STK17A binds to TRAF6 through its kinase domain (residues 112-120), engaging the TRAF domain (residues 358-364) of TRAF6. Although kinase-inactive STK17A mutants can bind TRAF6, binding of STK17A inhibits the auto-ubiquitination of TRAF6, thereby suppressing NF-kB-mediated inflammatory signaling 30.

#### Mitochondrial ANT2

Another important cytoplasmic binding partner is adenine nucleotide translocase 2 (ANT2), a mitochondrial inner membrane protein with well-established anti-apoptotic functions in cancer. ANT2 regulates mitochondrial permeability and catalyzes the exchange of ADP/ATP across the inner membrane. It promotes cell survival by inhibiting the pro-apoptotic Bax and by regulating the anti-apoptotic Bcl-2 and Bcl-xL. Upon cytoplasmic translocation to mitochondria, the catalytic domain of STK17A binds ANT2. Although it remains unclear whether STK17A directly phosphorylates ANT2, this interaction suggests that STK17A may modulate apoptosis by inhibiting ANT2. STK17A mutants that are constitutively retained in the cytoplasm display an even higher affinity for ANT2 10.

#### Myosin Light Chain (MLC)

STK17A has also been shown to phosphorylate myosin light chain (MLC), an exogenous substrate essential for cytoskeletal organization. This interaction was demonstrated in fibroblast-like monkey kidney cells (CO7 cells), where a kinase-defective mutant of STK17A failed to phosphorylate MLC. In mice fibroblasts (NIH3T3), overexpression of wild-type STK17A resulted in increased apoptosis and morphological changes 7. A similar role has been observed in Drosophila, where STK17 (DRAK) phosphorylates Spaghetti Squash (Sqh), the MLC ortholog, regulating cellularization and cytoskeletal dynamics 21.

## Physiological role of STK17A under non-pathological conditions

Despite the growing body of literature describing STK17A in disease contexts, its physiological function under normal conditions remains poorly defined. Unlike STK17B, whose roles are supported by genetic and *in vivo* models, current knowledge of STK17A is largely derived from *in vitro* studies and stress-induced systems. Available evidence suggests that STK17A may function as a stress-responsive kinase involved in cell fate regulation, including modulation of apoptosis, redox balance, and signaling homeostasis. However, whether STK17A plays an essential role in normal tissue maintenance or acts primarily as a context-dependent modulator activated under stress remains unclear. This knowledge gap represents an important limitation of the field and highlights the need for future studies using physiological and *in vivo* models to define the basal functions of STK17A.

## STK17A in Cancers

### STK17A Positive Outcomes in Cancers

STK17A has emerged as a significant regulator of apoptosis in a variety of cancers, where it often contributes to tumor suppression by promoting tumor cell death. Apoptosis in cancer cells can be initiated through intrinsic pathways (e.g., mitochondrial membrane permeabilization) and extrinsic pathways (e.g., death receptor activation by Fas ligand or TNF-α) 31. The extrinsic signaling pathway involves receptors from the tumor necrosis factor (TNF) receptor superfamily and are best characterized by the FasL/FasR and TNF-α/TNFR1 ligand models. The intrinsic signaling pathway is triggered by non-receptor stimuli, including growth factors deprivation, oxidative stress or radiation. These stimuli result in the release of pro-apoptotic factors from the mitochondria into the cytosol.

Among intrinsic mechanisms, the p53 signaling pathway is particularly significant. TP53 gene is mutated in most human cancers, contributing to tumor development. In healthy cells, p53 is targeted by the E3 ubiquitin ligase MDM2 for degradation. In response to stress such as DNA damage, p53 promotes G1 cell cycle arrest or mitochondrial permeabilization and subsequent apoptosis. Its downstream effectors include p21, which inhibits cyclin-dependent kinases to induce cell-cycle arrest; Noxa, which neutralizes the anti-apoptotic Bcl-2 proteins to promote cytochrome c release and caspase activation; and puma, which similarly promotes mitochondrial outer membrane permeabilization. Pro-apoptotic p53 targets such as Bax and Bad can also regulate the activity of mitochondrial ANT isoforms, thereby influencing mitochondrial apoptosis 10.

STK17A participates in multiple apoptotic pathways, but its role within the p53-dependent apoptotic cascade is particularly well established.

#### Testicular Cancer and Osteosarcoma

In testicular cancer, treatment with cisplatin — a DNA-damaging chemotherapeutic — upregulates STK17A expression in a dose-dependent manner, independently of STK17B. Similar observations have been made in osteosarcoma cell lines, where cisplatin induces both STK17A expression and p53 phosphorylation at Ser392, enhancing p53's transcriptional activity 10. Silencing p53 or STK17A markedly reduces sensitivity to cisplatin, while STK17A overexpression increases ROS production and promotes apoptosis 25.

Moreover, STK17A upregulation is not limited to cisplatin: vinblastine, doxorubicin, and etoposide also induce STK17A expression, indicating that STK17A responds broadly to genotoxic stress.

#### Prostate and Adenocarcinoma Cells

STK17A expression can be induced in the context of dsRNA and type I interferon (IFN) signaling, consistent with engagement of antiviral stress pathways. Current data support association with IFN/RNase L-linked apoptosis in specific cell-line models, but do not establish a causal role in virus-driven tumorigenesis**.** In prostate cancer and adenocarcinoma cell lines, the combination of dsRNA and IFN induces STK17A expression, which correlates with enhanced apoptosis via JNK activation and mitochondrial apoptotic pathways 27.

#### Cervical Cancer

In cervical cancers, STK17A exhibits anti-inflammatory properties. By binding to and destabilizing TRAF6, STK17A impairs the NF-kB pathway and reduces the expression of pro-inflammatory cytokines such as IL-1β, IL-8, and CXCL1 and CXCL2. Functionally, STK17A silencing enhances proliferation and invasiveness in HeLa and CaSki cervical cancer cell lines, further confirming its tumor-suppressive role 30.

#### Colon and Ovarian Cancers

In colon carcinoma, oxaliplatin-resistant cell lines exhibit downregulation of STK17A, while anti-apoptotic genes are upregulated 32. Similarly, in ovarian cancer (OVCAR3 cell line), STK17A overexpression does not trigger apoptosis per se but significantly sensitizes cells to chemotherapeutic agents such as carboplatin and paclitaxel. Conversely, STK17A knockdown enhances chemoresistance 33.

### STK17A Negative Outcomes in Cancers

Although STK17A is traditionally linked to apoptosis and tumor suppression, it paradoxically drives tumor progression and therapy resistance in certain cancers.

#### Glioblastoma

In glioblastoma, STK17A exhibits a pro-tumorigenic effect with expression levels increasing in a grade-dependent manner and correlating with malignancy. Silencing STK17A in glioblastoma cell lines (U87 and U251) reduces proliferation, migration, and invasion. Conversely, STK17A overexpression enhances cell survival and resistance to genotoxic stress, but does not significantly increase proliferation or migration, suggesting that while STK17A contributes to cell motility, it is not the primary driver 28.

In glioblastoma and low-grade of glioma datasets, STK17A expression has been reported to correlate with cytoskeletal regulators (e.g., MRLC, anillin) and with EGFR expression. In glial tumorigenesis, EGFR is overexpressed and PI3K mutated (active constitutive). STK17A could be a downstream of EGFR/PI3K and its co-overexpression with EGFR can promote cell proliferation and glial EGFR-PI3K-driven neoplasia, by directly phosphorylating MRLC at Thr^18^ and Ser^19^ which regulates changes to the cytoskeleton by promoting aniline binding to Zipper proteins 21.

#### Head and Neck Squamous Cell Carcinoma (HNSCC)

In HNSCC, STK17A is overexpressed at both the mRNA and protein levels and exerts a pro-tumoral role. It binds competitively to Smad3 in the cytoplasm, preventing its association with Smad4 and subsequent nuclear translocation. This inhibits TGF-β1 tumor suppressor signaling, which normally induces cell cycle arrest and restricts epithelial tumor progression. STK17A overexpression decreases TGF-β1 signaling and promotes tumor cell proliferation, whereas STK17A silencing restores TGF-β1 activity and reduces tumor cell proliferation 29.

#### Gastric Cancer

In advanced gastric cancer, STK17A expression is significantly upregulated and is associated with poor prognosis. Overexpression of STK17A in gastric cancer cells (MKN45 and SNU1) promotes proliferation, migration, and colony formation. It also induces epithelial-mesenchymal transition (EMT) markers, increasing N-cadherin and vimentin levels while decreasing E-cadherin expression — key features that facilitate metastasis 34.

### *In silico* Analysis

We analyzed STK17A mRNA expression across various cancer types using data from The Cancer Genome Atlas (TCGA) and compared tumor versus normal tissues usings GEPIA software (http://gepia.cancer-pku.cn). These analyses describe transcript-level associations and are limited by the absence of direct evidence for mechanistic causality or kinase activity.

**Figure 5:**
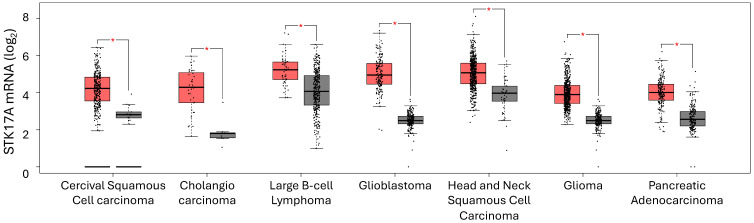
** Cancers exhibiting STK17A mRNA overexpression** (GEPIA). Red: STK17A mRNA level in tumor. Grey: STK17A mRNA level in non-tumoral tissue.

STK17A is overexpressed in several cancer types, including cervical cancer, colorectal cancer, diffuse large B-cell lymphoma, glioblastoma, head and neck squamous cell carcinoma, glioma, and pancreatic adenocarcinoma (**Figure 5**).

We subsequently investigated the association between STK17A overexpression and overall survival (OS) across all cancer types in the TCGA dataset using the cBioPortal software https://www.cbioportal.org/. High STK17A expression correlates with longer overall survival (OS) in melanoma and showed a favorable trend in cervical and colorectal cancers. Previous studies have reported an anti-tumor role for STK17A in cervical and colorectal cancers, although its function in melanoma remains to be elucidated.

Conversely, STK17A overexpression was associated with a shorter OS in glioma, head and neck squamous cell carcinoma, and gastric cancer. These findings are consistent with existing literature supporting a pro-tumor role of STK17A in these malignancies.

### Possible Explanations for the Dual Role of STK17A

The divergent roles of STK17A across cancer types are likely highly context-dependent (**Table 1**) and reflect differences in (i) baseline expression/addiction, (ii) dominant upstream signaling networks, and (iii) subcellular localization and interactome. Basal abundance may set the stage for whether STK17A is primarily engaged as a stress-effector (low baseline, inducible) versus a pathway “node” co-opted by oncogenic signaling (high baseline, constitutive). STK17A can be induced by genotoxic stress (cisplatin, vinblastine, doxorubicin, etoposide) and acts within a p53-linked apoptotic program, where STK17A supports ROS production and apoptotic sensitivity; in such settings, p53/STK17A silencing reduces chemotherapy responsiveness, consistent with a tumor-suppressive function. Conversely, in tumors where growth-factor signaling dominates, STK17A may be integrated downstream of oncogenic pathways: in glioblastoma/glioma, STK17A is described as pro-tumorigenic, associated with grade, and functionally connected to EGFR/PI3K circuitry and cytoskeletal control via MRLC phosphorylation, supporting proliferation programs and resistance to stress. Similarly, in HNSCC, STK17A can blunt tumor-suppressive TGF-β signaling by sequestering Smad3 in the cytoplasm and preventing Smad3/Smad4 complex formation, thereby favoring proliferation. In gastric cancer, STK17A overexpression is linked to poor prognosis and induction of EMT-like changes, consistent with a pro-invasive role. Mechanistically, STK17A's localization switch (nuclear vs cytoplasmic/mitochondrial) and partner availability may further bias outcomes: STK17A can interact with TRAF6 to dampen NF-kB inflammatory outputs (tumor-suppressive context in cervical cancer) or engage mitochondrial/cytoskeletal partners such as ANT2 and MRLC, which could differentially tune apoptosis versus motility depending on cellular state. Finally, microenvironment-derived signals (e.g., the IFN/RNase axis) together with post-transcriptional regulatory mechanisms may rewire STK17A-associated signaling networks, helping to explain how the same kinase can function as a tumor suppressor in some lineages yet promote tumor progression in others.

In conclusion, STK17A can be conceptualized as a context-dependent signaling node rather than a classical oncogene or tumor suppressor. In apoptosis-competent settings with intact stress-response machinery, STK17A acts as an apoptosis amplifier or chemosensitizer. In contrast, in tumors dominated by oncogenic signaling and defective apoptotic checkpoints, STK17A may function as a non-oncogene dependency supporting survival, migration, or therapy resistance. These divergent outcomes likely reflect differences in baseline expression, localization, interactome, and signaling context.

## STK17A in Other Diseases

Beyond its roles in cancer, STK17A is involved in a wide range of non-oncological diseases, including cardiovascular, autoimmune, infectious, and neurological disorders.

### Organ Transplantation

Evidence linking STK17A to organ transplantation outcomes is limited to observational human tissue studies. In liver transplantation cohorts (human samples), STK17A protein expression has been described in bile canaliculi of healthy grafts, with reduced or altered staining patterns observed during chronic rejection. STK17A has been proposed as a potential early biomarker for liver graft rejection 35.

These findings are correlative and do not establish a functional role for STK17A in graft rejection or tolerance. No mechanistic studies or genetic models have yet validated STK17A as a driver of biliary injury or immune-mediated rejection, and its potential utility should therefore be considered exploratory and biomarker-oriented rather than causal.

### Autoimmune Diseases

In autoimmune conditions such as systemic lupus erythematosus (SLE), STK17A expression is altered. SLE is an autoimmune disease in which about 50% of patients develop nephritis and cerebral vasculitis. During active phases of SLE, STK17A expression is repressed, which may contribute to impaired DNA damage repair, a hallmark of SLE pathology 8. Genetic polymorphisms in STK17A are associated with increased susceptibility to the disease.

Nevertheless, associations between STK17A and autoimmune diseases are primarily supported by human genetic and transcriptomic studies. These observations are restricted to human cohorts and lack functional validation. Importantly, causal involvement of STK17A in autoimmunity has not been demonstrated *in vivo*, in contrast to STK17B, whose immune regulatory functions are supported by genetic mouse models. Sex-specific effects have not been systematically addressed and remain an important unresolved variable.

### Neurodegenerative Diseases

Alzheimer's disease is characterized by progressive neuronal loss and the accumulation of insoluble protein aggregates, such as amyloid beta, accompanied by secondary inflammatory and immune responses. In Drosophila models, STK17A orthologs have been implicated in the regulation of amyloid precursor protein (APP) metabolism, suggesting a possible protective role of STK17A against amyloid beta accumulation 36. These findings suggest evolutionary conservation of stress-related functions but cannot be directly extrapolated to human disease. No direct evidence currently demonstrates a role for STK17A in mammalian neurodegeneration, and reported associations should be interpreted as hypothesis-generating rather than mechanistic.

### Neurological and Psychiatric Disorders

Emerging evidence links STK17A to neurological and psychiatric conditions. STK17A is abnormally expressed in the brains of individuals with major depressive disorder, suggesting a potential involvement in psychiatric diseases 37. In chickens, STK17A polymorphisms have been correlated with anxiety-like behaviors, indicating a possible conserved role in stress response regulation across species 18. Nevertheless, as in neurodegenerative disease, there is no experimental evidence clearly demonstrating a direct link between STK17A and psychiatric conditions.

### Infectious Diseases

STK17A plays also a role in host defense against bacterial infections. In infections caused by *Mycobacterium tuberculosis*, *Mycobacterium avium*, or* Mycobacterium abscessus*, STK17A expression is upregulated in macrophages, promoting apoptosis and limiting pathogen replication 38,39. Genetic studies have shown that mutations on chromosome 7p13, where STK17A is located, reduce STK17A expression and increase susceptibility to non-tuberculous mycobacterial pulmonary disease 40. However, direct genetic or functional evidence demonstrated that STK17A is required for host defense, pathogen clearance, or apoptosis *in vivo* is currently lacking.

### Age/sex-related pathologies

Current evidence linking STK17A to age/sex-related pathologies remains limited and largely indirect. Transcript-level analyses of human bone marrow samples reported sex-associated increases in STK17A expression, particularly in females, with correlations to markers of osteoclast genesis and apoptotic regulation in age-related disorders such as osteoarthritis and rheumatoid arthritis, without demonstrating mechanistic causality. It also reported higher STK17A expression in osteoarthritis and rheumatoid arthritis patients compared with normal bone marrow cells 41. More recently, genome-wide association studies identified STK17A among shared genetic loci associated with age-related cataract and hearing difficulties 42. Collectively, these findings support a potential association between STK17A and aging-related tissue stress responses, while a direct causal role in age-related disease pathogenesis has yet to be established.

### Metabolic disorders (Diabetes mellitus)

Diabetes mellitus is a disorder characterized by metabolic anomalies marked by insulin resistance, relative insulin deficiency, and persistent hyperglycemia. During the progression of diabetes, STK17A may contribute to the advancement of the disease by regulating oxidative stress, programmed cell death pathways, and critical signaling pathways such as p53 and MAPK, thereby promoting the death of islet cells 43. Moreover, STK17A has been also discovered as a gene that can differentiate between individuals with obstructive sleep apnea and diabetes mellitus based on four machine learning methods. Indeed, chronic intermittent hypoxemia increases oxidative stress by enhancing relative oxygen production and oxidative/antioxidative imbalance in obstructive sleep apnea patients and oxidative stress permeates the development of diabetes and its complications 44. Although STK17A has been proposed to influence β-cell survival through stress-associated pathways, no direct functional or genetic evidence confirms its involvement in diabetes onset or progression.

## Comparison STK17A / STK17B

**Figure 8:**
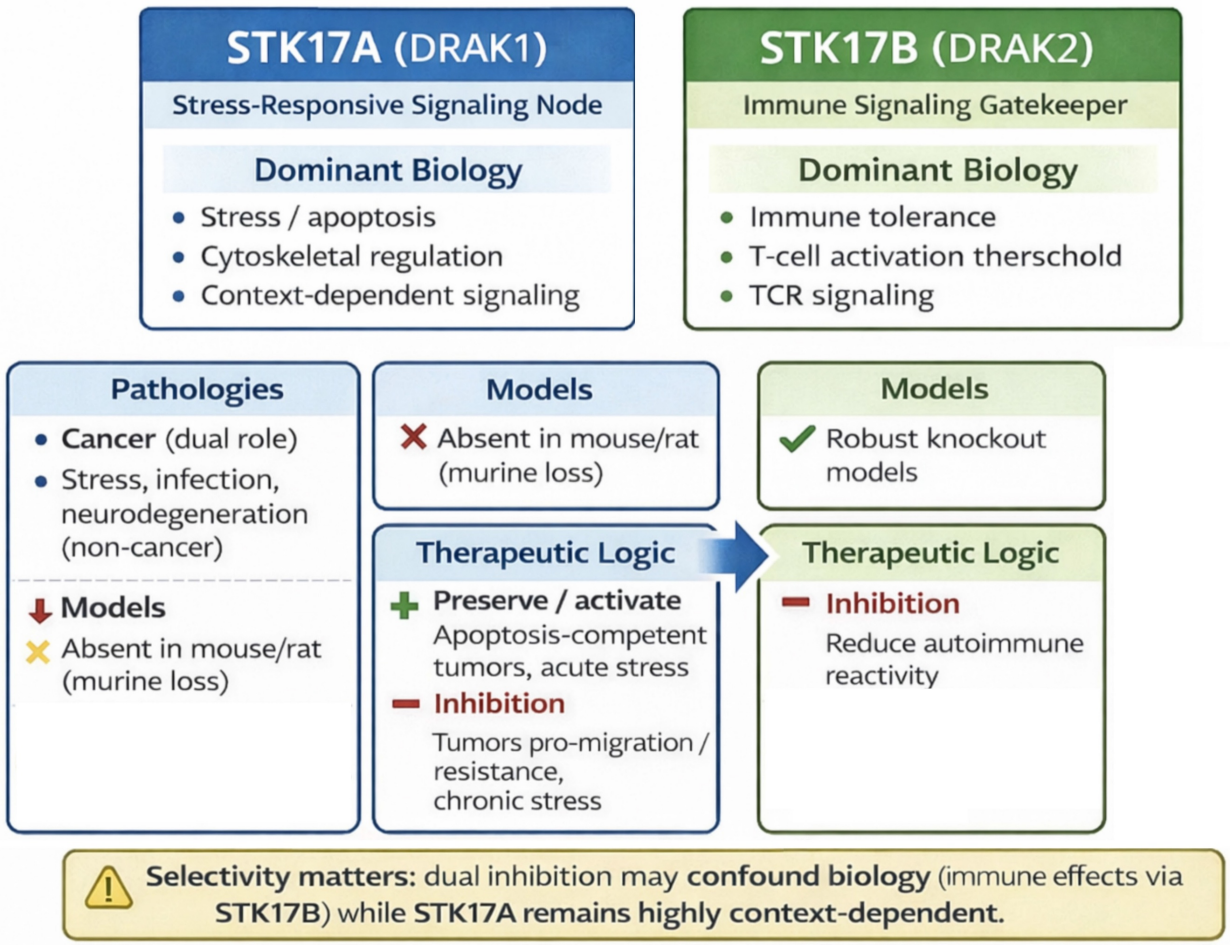
**Distinct biological roles and therapeutic implications of STK17A and STK17B**.

STK17B functions are supported by genetic and *in vivo* models—particularly in immune regulation—whereas STK17A evidence relies predominantly on cell-line perturbation and correlative analyses. This asymmetry has important implications for therapeutic prioritization: confidence in STK17A mechanisms and safety liabilities is necessarily lower until validated by improved models and selective chemical probes (**Table 2** and **Figure 8**).

STK17A and STK17B share a high degree of sequence similarity within their catalytic serine/threonine kinase domains, reflecting their common origin as members of the DAPK subfamily. Both proteins lack the CaM-binding domain characteristic of other DAPK family members and are primarily localized in the nucleus, where they contribute to the regulation of cell death pathways. Despite these structural and functional similarities, several differences distinguish between the two kinases. STK17B is predominantly expressed in lymphoid tissues and has been extensively implicated in immune cell homeostasis and tolerance. By contrast, STK17A shows a broader but generally lower expression profile and remains comparatively less studied. Emerging evidence suggests that STK17A may play a role in apoptosis and tumorigenesis, whereas STK17B has been more clearly associated with T cell signaling and autoimmune regulation. These observations highlight that, while structurally related, STK17A and STK17B may fulfill non-redundant, context-dependent biological functions.

### Role in T Cells and Immune Regulation

STK17B is predominantly expressed in lymphoid tissues and is highly enriched in B cells and T cells. It acts as a negative regulator of T cell activation, fine-tuning immune responses. During T cell development, STK17B expression fluctuates and is tightly regulated. STK17B deficiency results in T cell hypersensitivity to TCR (T cell receptor) stimulation, requiring less co-stimulatory signaling for activation 45. In STK17B knockout mice, T cells are hyperresponsive but surprisingly exhibit resistance to autoimmune diseases such as experimental autoimmune encephalomyelitis (EAE), a model for multiple sclerosis, and type 1 diabetes in NOD mice 46.

Conversely, overexpression of STK17B in transgenic mice enhances T cell apoptosis in an IL-2-dependent manner and impairs memory T cell development 47. Thus, STK17B plays a critical role in balancing T cell activation, survival, and memory formation. In comparison, STK17A is expressed in several tissues, but its role in T lymphocytes is less well characterized than that of STK17B 47.

### Interaction with TGF-β Signaling

Unlike STK17A, which binds to Smad3, STK17B inhibits TGF-β signaling through a different mechanism. It binds directly to the TGF-β type I receptor (TβRI), blocking the phosphorylation and activation of Smad2/3 48. Notably, STK17B can bind to both active and inactive forms of TβRI, distinguishing it from other negative regulators such as Smad7.

This interaction has been demonstrated in breast cancer, hepatocellular carcinoma, and cervical cancer cell lines, where STK17B overexpression suppresses TGF-β-induced tumor-suppressive signaling. Interestingly, despite its nuclear localization in many cancer cells, STK17B also localizes to the cytoplasm and membrane where it interacts with TβRI.

However, in T cells, STK17B does not inhibit TGF-β signaling, as Smad3/4 activation remains intact in wild-type and STK17B knockout T cells 49.

### Tumorigenic and Tumor Suppressor Roles

STK17B displays context-dependent effects in cancers. In breast cancer, STK17B overexpression promotes tumor progression whereas in myeloid leukemia and in colorectal cancer, STK17B acts as a tumor suppressor, enhancing apoptosis when overexpressed. In myeloid leukemia models, oncogene silencing de-repressing STK17B leads to increased apoptosis 50.

Interestingly, despite these *in vitro* effects, STK17B knockout mice show no difference in spontaneous tumor development, even when crossed with p53-deficient mice or exposed to carcinogens, suggesting that STK17B alone may not play a dominant role in tumor surveillance *in vivo* 50.

Compared to STK17A, the role of STK17B in cancer is less extensively characterized; however, both kinases exhibit distinct and context-dependent functions across different tumor types.

### Role in Neuronal Development

STK17B also phosphorylates myosin-II regulatory light chain (MRLC), like Rho kinases, influencing cytoskeletal dynamics. In neurons, STK17B inhibits axon outgrowth, elongation, and branching. Pharmacological inhibition of STK17B with compounds such as SC82510 enhances axonal regeneration, suggesting that STK17B acts as a negative regulator of neuronal regeneration 51.

### Conclusion

The biological complexity and context-dependent roles of STK17A and STK17B underscore their relevance as signaling nodes in apoptosis, immunity, cytoskeletal control and tumorigenesis.

However, despite growing insights into their molecular interactions and disease associations, the precise mechanisms governing their activity remain incompletely understood. Indeed, major limitations constrain current interpretation for STK17A: (i) reliance on overexpression/knockdown systems; (ii) limited validation of endogenous substrates and kinase activity; (iii) absence of murine genetic models; and (iv) frequent overinterpretation of transcriptomic or network associations as mechanistic causality. Future work should prioritize (a) development and broad dissemination of selective, cell-active STK17 chemical probes; (b) quantitative phosphoproteomics and orthogonal substrate-validation pipelines and (c) alternative *in vivo* models (e.g., zebrafish or humanized systems) that enable genetic interrogation.

Particularly, the development of small-molecule inhibitors would not only provide essential probes to clarify the distinct and overlapping roles of STK17A and STK17B, but also offer opportunities for therapeutic intervention in cancers, autoimmune, and neurodegenerative diseases. Recent medicinal chemistry efforts have, therefore, focused on designing scaffolds with improved potency and selectivity on each STK17 kinase, paving the way for both mechanistic studies and potential clinical applications.

## STK17A/B Inhibitors

Despite the therapeutic promise of STK17A, only a few STK17A inhibitors have been reported to date 9. Conversely, inhibitors of STK17B have attracted more attention, particularly for the treatment of autoimmune diseases and the prevention of transplant rejection 52. Although this review primarily focuses on STK17A, the identification of inhibitors targeting both STK17A and STK17B is of significant interest for advancing our understanding of their biological functions and therapeutic implications, as well as for the development of novel pharmacological agents. The discovery of new, potent, and truly selective chemical scaffolds could serve as a foundation for further optimization in drug discovery and provide valuable insights into the distinct and overlapping roles of these kinases in biological systems.

Notably, the development of STK17A-selective inhibitors remains a critical unmet need. Such compounds would enable precise dissection of STK17A-specific signaling pathways and functions while minimizing unintended interference with STK17B-mediated immune regulation. Beyond their utility as mechanistic probes, selective STK17A inhibitors could offer safer and more effective therapeutic strategies in cancers and non-oncological diseases in which STK17A plays a pivotal role.

The first STK17 inhibitor report is a STK17B inhibitor (SC82510, small molecule with undisclosed structure) described only in 2014 51. It exhibits a low micromolar IC_50_ yet promotes *in cellulo* activity at 1-5 nM, suggesting that significant off-target effects may be expected. SC82510 is an improved derivative of 2-(2-(trifluoromethoxy)benzamido)-N-(6-(trifluoromethyl)-1H-benzo[d]imidazol-2-yl)thiazole-4-carboxamide, another kinase (CK1δ/ε) inhibitor 53. SC82510 had a much cleaner profile in the KINOMEscan™ assay, inhibiting only STK17A, STK17B and RPSK2, although less active than the other analogues tested. Interestingly, inactive compounds resulted from a benzothiazole core versus a benzimidazole one. Furthermore, the inhibition of STK17B in cells was associated to axon outgrowth, proposing a pro-regenerative activity with potential therapeutic applications following axonal lesions 51.

**Figure 9:**
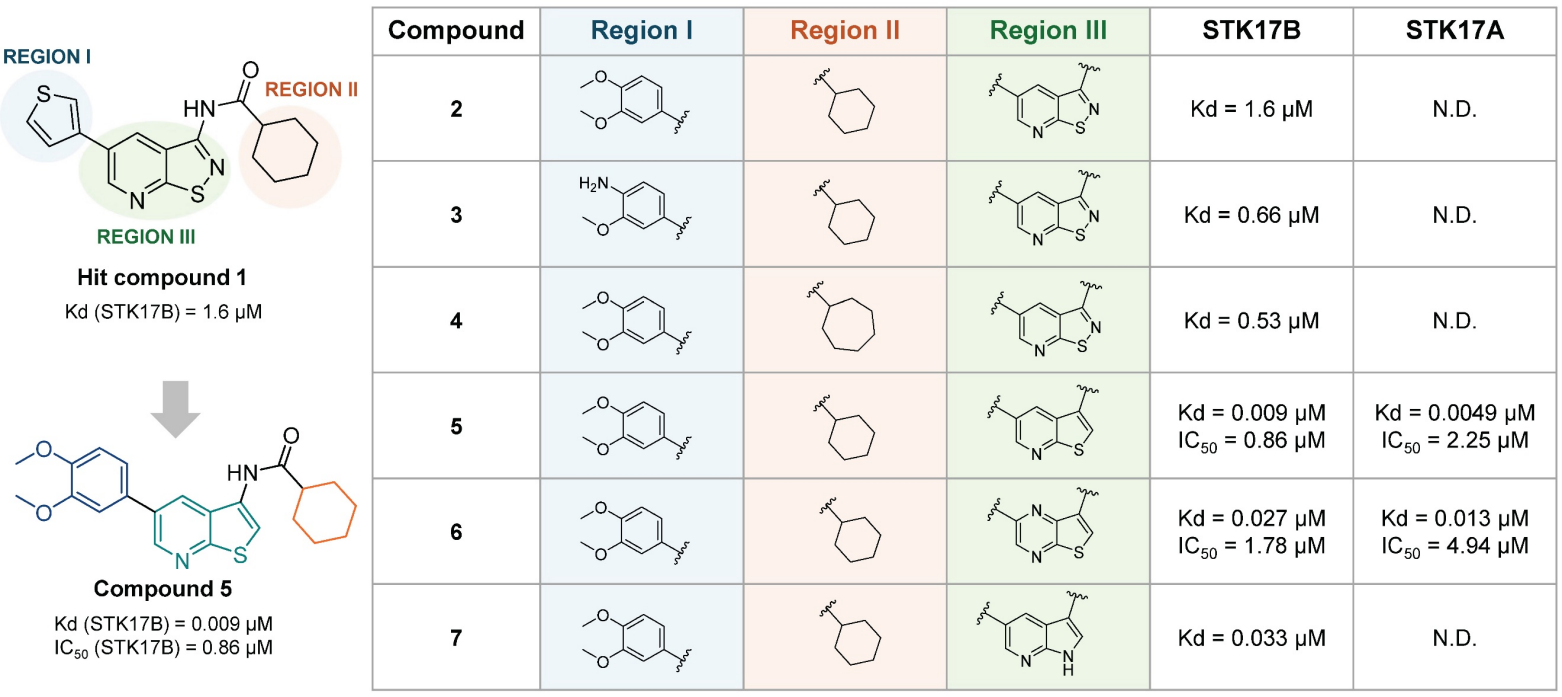
**Discovery of thieno[2,3-b]pyridine derivatives as potent STK17A/B ligands** 52**.** Representative compounds with corresponding binding constants (Kd) and enzymatic activity IC_50_ values.

In the same year, Gao et al. reported an isothiazolo[5,4-b]pyridine backbone (**1, Figure 9**) as novel STK17B ligand (Kd = 1.6 µM) discovered from a 150 compounds library screening in the DiscoverX binding assays 52. The optimization of this structure towards affinity enhancement led to thieno[2,3-b] pyridine derivatives, revealing compound **5** (**Figure 9**) as a dual STK17A (Kd = 5 nM, IC_50_ = 2.25 µM) and STK17B (Kd = 9 nM, IC_50_ = 0.86 µM) inhibitor. Modifications of the two regions, namely 5-aryl (region I, **Figure 9**) and cyclohexyl (region II, **Figure 9**), did not lead to affinity improvement, with the best compounds as **2**, **3**, and **4** (**Figure 9**). Besides, the replacement of cycloalkyl amides by non-cylic and aromatic amides led to complete loss of STK17B binding. Scaffold hopping on the isothiazolopyridine scaffold led to compound **5** with the highest affinity for STK17B (Kd = 9 nM) and compound **6** with Kd = 27 nM (**Figure 9**). Nevertheless, both compounds were not selective to STK17B, showing also strong STK17A binding (Kd = 4.9 nM and 13 nM, respectively). Functional activity against both STK17A and STK17B enzymes confirmed the dual inhibitory properties of compounds **5** and **6**, albeit with IC₅₀ values in the micromolar range. The difference in affinity and activity values remains unclear, despite the suggestion that these compounds act as ATP competitive inhibitors might partially account for it. Interestingly, compound **7** shows strong affinity for STK17B but lacks completely functional inhibitory activity.

**Figure 10:**
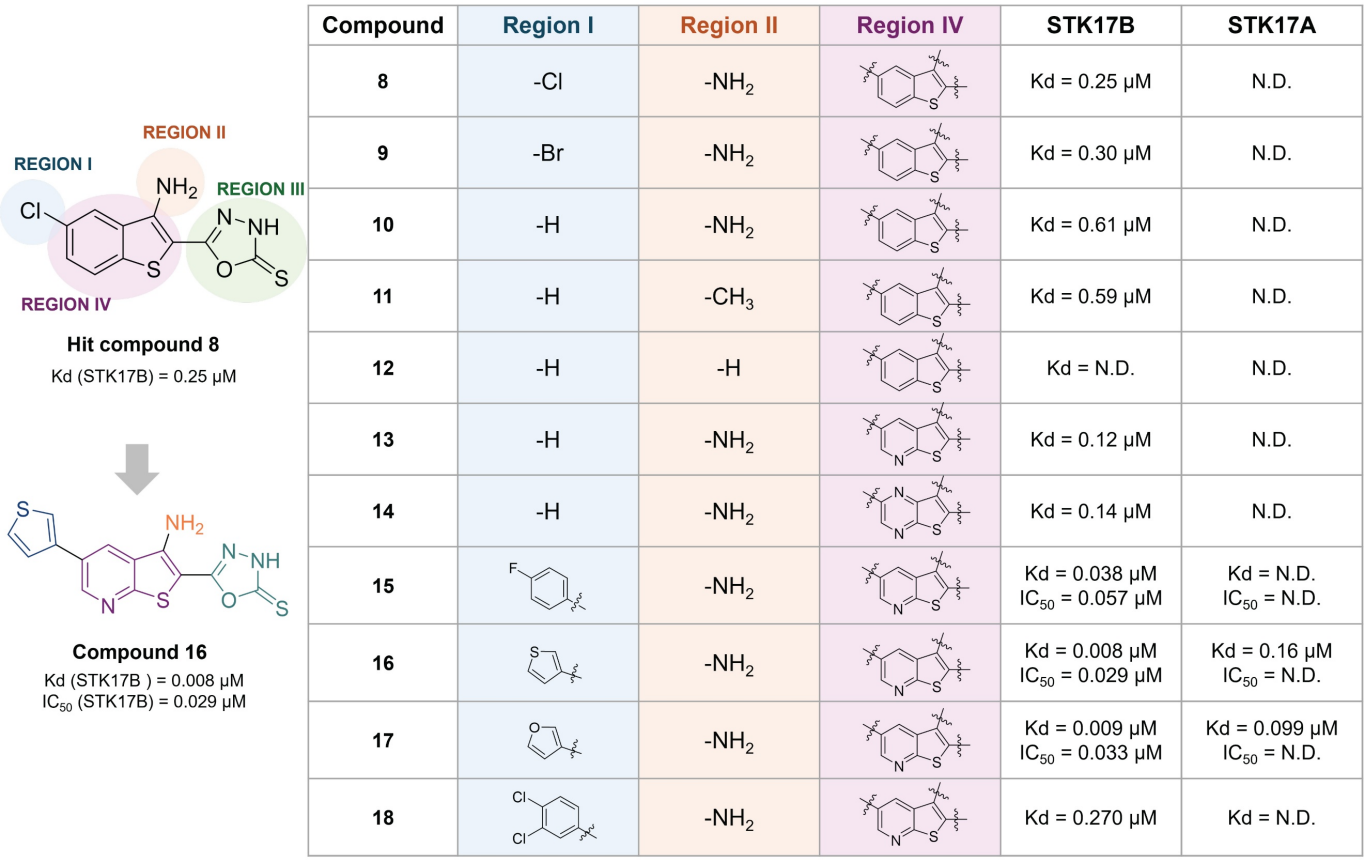
** Optimization of thieno[2,3-b]pyridine scaffold as potent non-selective STK17B ligand** 54. Representative compounds with corresponding binding constants (Kd) and enzymatic activity IC_50_ values.

The same research group reported a benzothiophene hit (**8**) with high affinity to STK17B (Kd = 0.25 µM), optimized towards compound **16** (**Figure 10**) 54. This optimized inhibitor still shows low selectivity within the DAPK family but shows improved inhibitory activity for STK17B (Kd = 8 nM, IC_50_ = 29 nM, **Figure 10**) than the previous developed compound **5** (Kd = 9 nM, IC_50_ = 860 nM, **Figure 9**) 52. SAR was conducted based on the modifications of 4 regions (**Figure 10**). The substitution of the phenyl ring with electron-withdrawing and electron-donating substituents did not afford better analogues than the hit compound **8**. The presence of the halogen (compound** 8**, **9** vs **10**) in position 6 and of the amino in position 3 (compound** 10** *vs* **11**) only showed a slight beneficial effect (**Figure 10**). In contrast, the thioxo group in the oxadiazole moiety in region III was revealed to be essential for improving activity. The modulation of the benzothiophene (region IV) by introduction of nitrogen atoms in the 6-membered ring (compound** 13**, **14**) resulted in compounds with 5-fold tighter binding than the hit compound **8**. Therefore, the thieno[2,3-b]pyridine scaffold was selected for further optimization of region I. Introduction of aryl substituents in position 6 resulted in drastic binding improvements with low nanomolar Kd values. In addition, the 3-thiophenyl and 3-furanyl derivatives **16** and **17** demonstrated high enzymatic activity (IC_50_ = 29 nM and 33 nM, respectively, **Figure 10**). However, the selectivity of these compounds remained limited, with compound **18** showing the highest selectivity although with lower activity.

**Figure 11:**
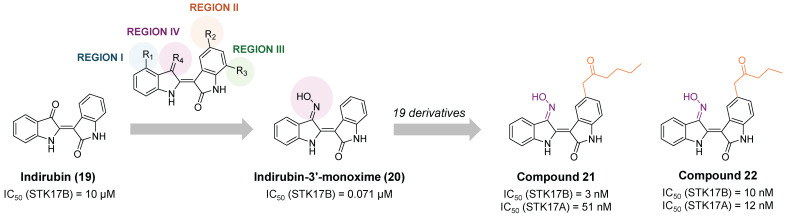
**Optimization of indirubin toward potent indirubin-3'-monoxime non-selective STK17A/B ligands** 36**.** Representative compounds with corresponding enzymatic IC_50_ values.

In 2016, indirubin derivatives were identified as a promising scaffold for STK17B inhibition on a high throughput screening 55. Optimization of the hit compound indirubin (compound **19, Figure 11**) —the active component of a traditional Chinese medicinal formulation, *Danggui Longhui Wan*—revealed that the oxime group at R4 position and small aliphatic groups at R5 are critical for enhancing STK17B inhibitory activity. Derivatives of indirubin-3'-monoxime (compound **20**) led to the discovery of compound **21** as the most potent derivative with an inhibitory value for STK17B IC_50_ = 3 nM and Ki = 0.25 nM. Interestingly, compound **22**, bearing a propylketone (region II) instead of the butylketone (compound **21**), exhibited high activity against both STK17B and STK17A (**Figure 11**). Enzyme kinetics and molecular docking studies indicated that compound **21** is an ATP-competitive inhibitor. Although all analogues exhibited moderate inhibitory activity against DAPK1-3 and strong inhibition for STK17A, compound **21** demonstrated the highest selectivity. Docking studies were consistent with the higher binding affinity observed for STK17B compared to other DAPK family members, attributing this selectivity to the interaction of the inhibitor with the active site residue Tyr112.

**Figure 12:**
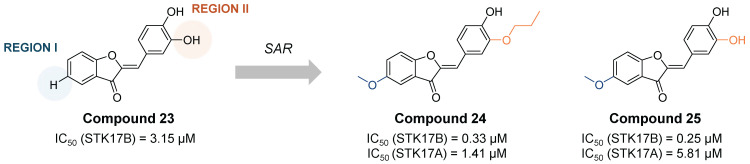
**Optimization of benzofuran-3(2H)-one non-selective STK17B inhibitors** 56**.** Representative compounds with corresponding enzymatic IC_50_ values.

Another class of STK17B inhibitors presenting a benzofuran-3(2H)-one scaffold was discovered on a high throughput screening 56. From the hit compound **23** (IC_50_ STK17B = 3.15 µM, **Figure 12**), the SAR study let to the obtention of compounds **24** and **25** (**Figure 12**) with IC_50_ values around 10-fold lower than the initial hit (IC_50_ STK17B = 0.33 µM and 0.25 µM, respectively). Although both compounds are nonselective STK17B inhibitors with residual activities against other members of the DAPK family, including STK17A, compound **24** showed a more favorable selectivity profile than compound **25** against other kinases of the DAPK family. Both compounds were found to protect islet b-cells from apoptosis in dose dependent manners, supporting the hypothesis that STK17B inhibitors might be a promising approach for the treatment of diabetes.

**Figure 13:**
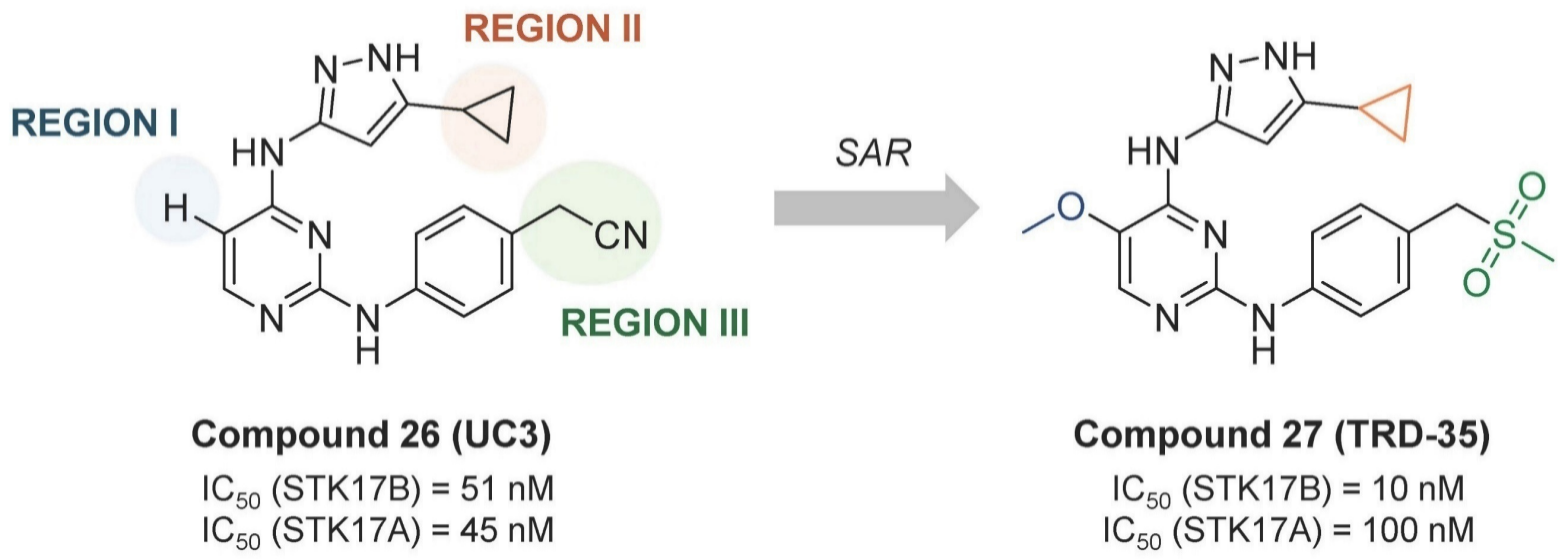
**Optimization of promiscuous 3-aminopyrazoylpyrimidine (compound 26, UC-3) toward potent and selective STK17B inhibitor compound 27 (TRD-35)** 57. Representative compounds with corresponding enzymatic IC_50_ values.

From the promiscuous kinase inhibitor (compound **26**, UC-3, IC_50_ STK17B = 51 nM), Ali et. al. developed a promising quite selective 3-aminopyrazoylpyrimidine STK17B inhibitor (compound **27**, TRD-35, IC_50_ STK17B = 10 nM), though no cellular activity is reported (**Figure 13**) 57. Interestingly, the replacement of the cyclopropyl by *tert*-butyl led to total loss of activity (IC_50_ > 10000 nM).

**Figure 14:**
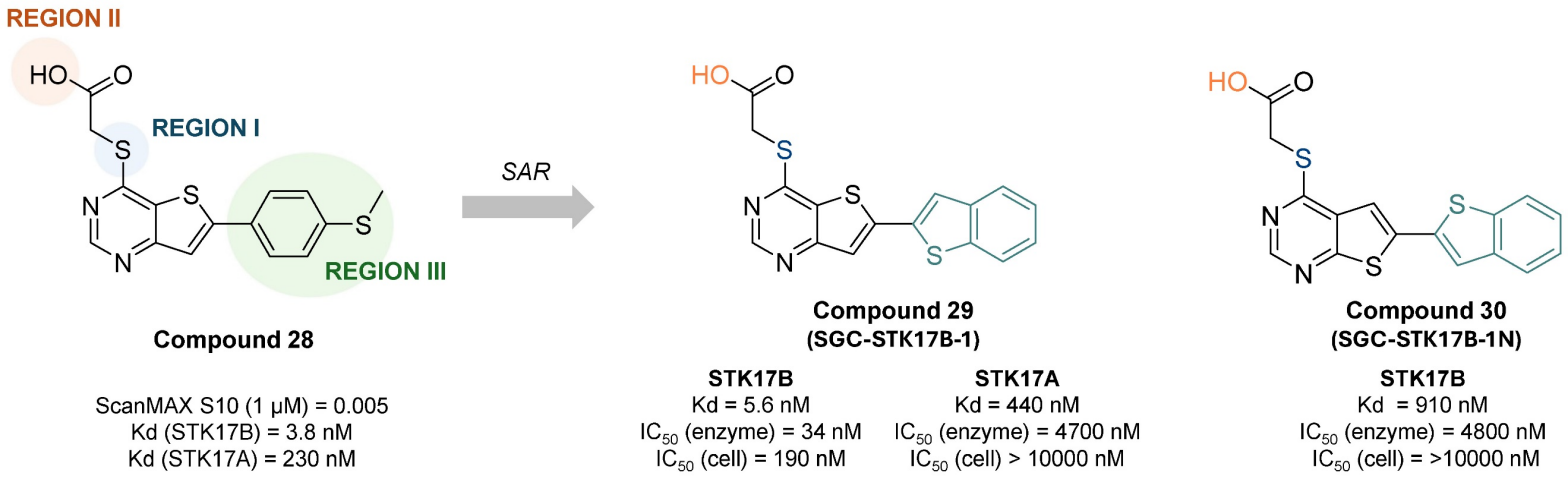
**Discovery of thieno[3,2d] pyrimidines as selective and potent STK17B inhibitors.** Representative compounds with corresponding binding constants (Kd) and activity IC_50_ values. ScanMAX, S_10_ (1 μM) = number of kinases with 10% or less of control activity remaining at 1 μM / number of wild type kinases tested (403), n = 1. Kd determined by dose response, n = 2. IC_50_ for enzyme inhibition at Km ATP determined at Eurofins, n = 2. Target engagement IC_50_ determined by NanoBRET assay in HEK293 cells 58.

The most selective and *in cellulo* active STK17B inhibitor reported to date was described by Picado et al. in 2020 58. Thieno[3,2d] pyrimidines were screened in the DiscoverX scanMAX panel screen against 403 wild-type human kinases leading to the discovery of PFE-PKIS 43 (compound** 28**, **Figure 14**). They also revealed by MD simulations that a unique P-loop conformation appears to be the molecular basis of their remarkable potency and selectivity. SAR studies demonstrated the critical need of the sulfur atom in region I as well as the carboxylic acid in region II for activity. Extensive SAR study on region III led to optimized compound **29** (SGC-STK17B-1, IC_50_ STK17B = 34 nM) with high selectivity and potency contrary to its isomer **30** (SGC-STK17B-1N, IC_50_ STK17B = 4800 nM). The drastic loss in activity for compound **30** was attributed to the reduced basicity of the pyrimidine N1, which does not support a similar strength of molecular recognition.

**Figure 15:**
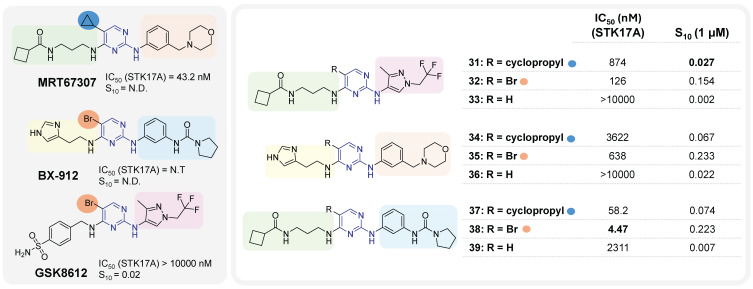
**Aminopyrimidines derivatives and corresponding STK17A IC_50_ values (NanoBRET cell assay) and selectivity indexes S_10_** 59. N.D.: not determined. S_10_ (selectivity index at 1μM): percentage of screened kinases with percentage of control values < 10% at 1μM. The lower the S_10_ value, the higher the selectivity.

It was only from 2021 onwards that studies focusing on the development of selective STK17A inhibitors began to emerge. In that year, Drewry at al. identified six 2,4-diaminopyrimidine-based compounds as non-selective submicromolar STK17A inhibitors, in the search of ligands for understudied kinases implicated in driving neurodegeneration 59. These pyrimidine-based derivatives were developed by diversely substituting both amino groups. This strategy can lead to an additional hydrogen bond with the kinase conserved hinge region found in nearly all human kinases along with the key hydrogen bond associated with nitrogen of the pyrimidine scaffold, extensively used in the design of kinase inhibitors. The derivatives were designed based on the structure of three previously studied TBK1 inhibitors **MRT67307**, **BX-912** and **GSK8612** (**Figure 15**). The mixing and matching of the side chains and cores from these three inhibitors led to 21 derivatives 59. A first kinase panel screening (14 kinases, Eurofins) demonstrated the enzymatic activity of some compounds towards STK17A, which was subsequently confirmed through single-dose and dose-response assays using NanoBRET. Compounds **31**,** 32**,** 35**,** 38**,** 37** and **MRT67307** (**Figure 15**) were shown to have submicromolar activity in the NanoBRET STK17A assay. Interestingly, substitution in position 5 of the pyrimidine (-R, **Figure 15**) appeared critical for activity, with bromine as the best substituent. Compounds **35**, **34**,** 38**, and** 37** also showed submicromolar inhibitory activities against MARK4 kinase in NanoBRET, whereas compounds **35** and **38** showed submicromolar activities against MARK3. Overall, compound **31** may represent the most promising molecule of this series, despite its moderate activity (S_10_ = 0.027, IC_50_ = 874 nM,** Figure 15**).

**Figure 16:**
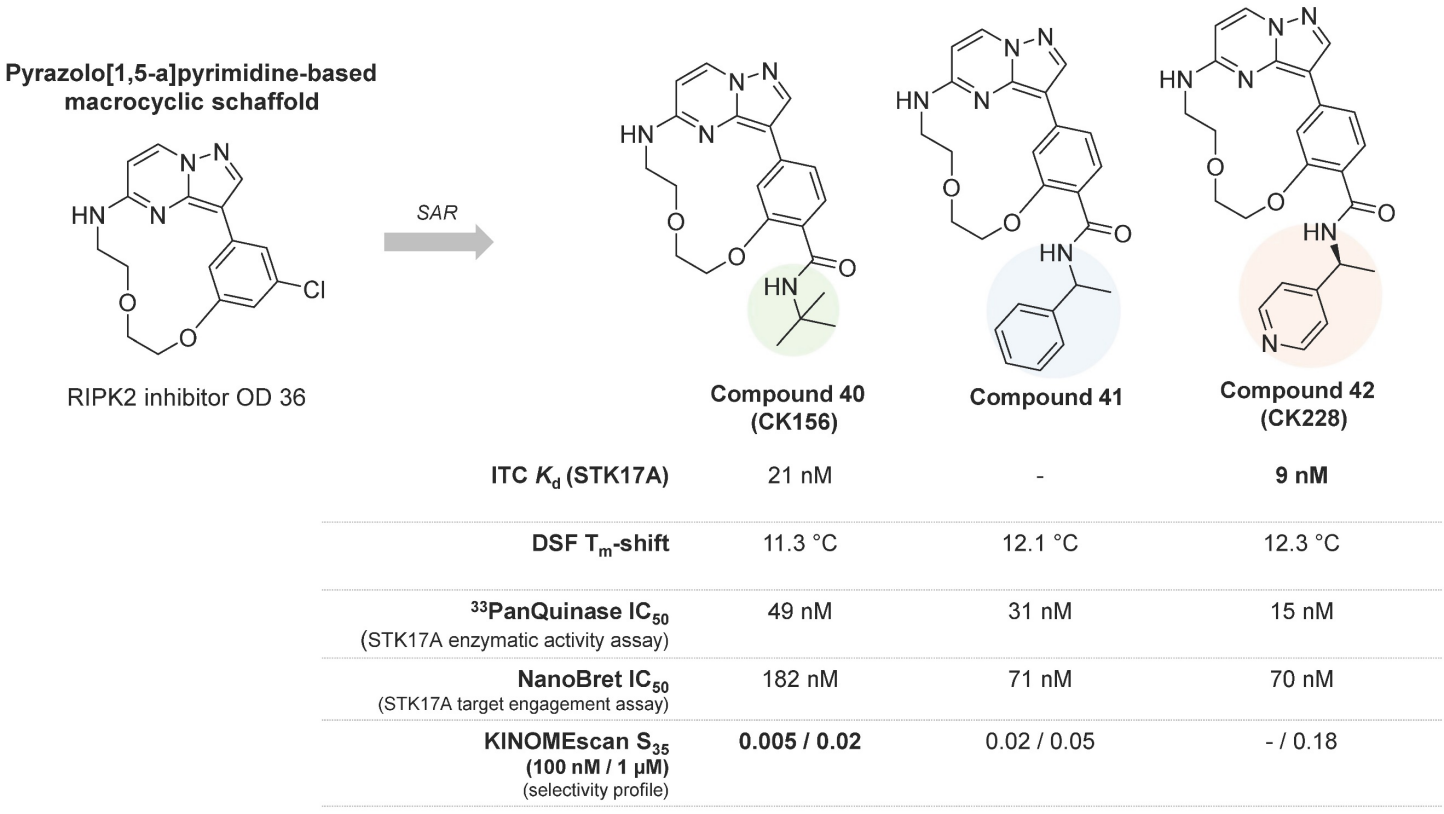
** Macrocyclic kinase inhibitors and their activities against STK17A determined using various analytical techniques** 60**.** DSF: differential scanning fluorimetry; ITC: isothermal titration calorimetry; ^33^PanQuinase (Reaction Biology): enzymatic activity assay; NanoBRET (Promega): target engagement assays; KINOMEscan (Eurofins Discovery): selectivity profile. S_35_: selectivity index score. The S-score quantifies the selectivity at the measured concentration; threshold of %Ctrl = 35% [S_35_ = (number of non-mutant kinases with %Ctrl <35)/ (number of non-mutant kinases tested)]. The lower the S_35_ value, the higher the selectivity.

In 2022, Kurz et. al. reported the first selective STK17A inhibitor (CK156, compound **40**, **Figure 16**), exhibiting high *in vitro* potency (*K*_d_ = 21 nM, IC_50_ = 49 nM) and remarkable selectivity in kinome widescreens (S_35_ = 0.005 at 100 nM and S_35_ = 0.02 at 1 mM; S_35_: selectivity score) 60. This achievement was permitted by structure-guided optimization of a pyrazolo[1,5-a]pyrimidine-based macrocyclic scaffold (**Figure 16**), previously found as a RIPK2 inhibitor 61. Macrocyclic kinase inhibitors typically contain an ATP-mimetic pharmacophore, which is constrained by a linker of 12 or more atoms, connecting flexible moieties of acyclic conventional inhibitors 62.

The optimization of the pyrazolo[1,5-a] pyrimidine-based macrocyclic scaffold resulted in better potent inhibitors of STK17A, compounds **41** and **42** (**Figure 16**), with *in vitro* activity values of 31 nM and 15 nM, respectively 60. Nevertheless, compounds **41** and **42** showed lower selectivity in the KINOMEscan (high throughput site-directed competition affinity binding assay over 500 kinase domain-containing human wildtype and clinically relevant mutant targets) in comparison to compound **40** (**Figure 16**), which showed exclusive affinity to STK17A, with an IC_50_ value of 182 nM. Indeed, affinities for other potential off-target kinases were 30-fold lower compared to STK17A. The first crystal structure of STK17A was obtained through co-crystallization with compound **40**, revealing a classical type I inhibitor binding mode. The *tert*-butyl group of compounds **40** was observed to point to the back pocket of STK17A and does not disturb key H-bonds with the hinge region and Lys90. In addition, cell viability studies showed no toxicity of compound **40** in HEK293T, U2OS, and MRC-9 cells (1 μM concentration). Despite the unknown *in vivo* activity and bioavailability of the pyrazolo[1,5-a] pyrimidine-based STK17A inhibitors, compound **40** is a very promising chemical probe and provides valuable insights for the development of selective STK17A inhibitors.

**Figure 17:**
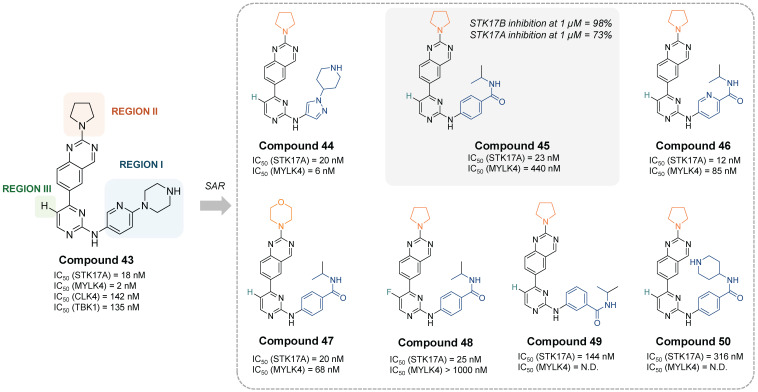
**Quinazoline-based STK17A/B inhibitors and their activities** 63**.** IC_50_ values were based on enzyme activity assays with an ATP concentration of 10 μM (Reaction Biology Corporation). STK17B inhibition was tested only with compound **45** (1 µM). CLK4 IC_50_ is not included in the figure because it is either > 10,000 nM or not determined.

In the most recent work, Chaudhry, S. presented a quinazoline-based STK17A/B dual inhibitors with good drug-like profile and oral bioavailability 63. The developed inhibitors selectively target STK17A and STK17B and were shown to be effective in viability assays against cancer cell lines, suggesting a role of STK17A as a therapeutic kinase target in various cancers. Compound **43** was the first hit in a profiling study against a panel of 375 kinases. It showed strong inhibition of MYLK4 (IC_50_ = 2 nM) and STK17A (IC_50_ = 18 nM), and moderate potency against TBK1 (IC_50_ = 135 nM) and CLK4 (IC_50_ = 142 nM). The SAR study over 19 analogues revealed that the modifications in the region I with an *N*-piperidine pyrazole moiety (compound **44**, IC_50_ = 20 nM) or a 4-yl-isopropylbenzamide moiety (compound **45**, IC_50_ = 23 nM) led to derivatives with STK17A inhibitory potency comparable to that of compound **40** (**Figure 16**). Yet, better potency was also observed when the aromatic ring in Region-I was a pyridine instead of a benzene moiety (compound **46**, IC_50_ =12 nM). Nevertheless, neither the modification of region II by a morpholine ring (compound **47**, IC_50_ = 20 nM) nor the modification of region III by a F-substitution (compound** 48**, IC_50_ = 25 nM) showed significant improvement (**Figure 17**). Besides, reduction on the STK17A inhibition was observed with the 3-yl-isopropylbenzamide moiety (compound **49**, IC_50_ = 144 nM) and further with the 4-yl-piperidinebenzamide moiety (compound **50**, IC_50_ = 316 nM), probably due to the additional NH that could form H-bonds with surrounding protein residues. The selectivity against MYLK4 and CLK4 kinases was evaluated, revealing that compounds **45** and **48** were the most selective (**Figure 17**). Both compounds also displayed favorable *in vitro* DMPK properties, including good human/mouse microsomal stability and low inhibition of cytochrome P450 isoforms. Compound **45** was selected as the lead candidate, and *in silico* studies suggested a Type-I kinase inhibition mode. However, final selectivity assessment of compound **45** toward STK17A showed 98% inhibition of STK17B versus 73% inhibition of STK17A at 1 µM, indicating that compound **45** acts as a dual STK17A/B inhibitor with greater potency against STK17B. Biological evaluation of compound **45** across three acute myeloid leukemia (AML) cell lines (MOLM-13, HEL, and Kasumi-1) revealed EC₅₀ values in the low micromolar range, while demonstrating good selectivity over the immortalized cell line HEK-293T (IC_50_ ≈ 100 μM). However, direct target-engagement evidence supporting this AML activity remains to be established. Pharmacokinetics studies in C57BL/6 mice after oral administration (vehicle: 2% Tween80 / 10% DMSO / 88% PBS) of compound **45** (dose: 10 mg/kg) indicated a reasonable exposure (t_1/2_ = 2.7 h, T_max_ = 1h, C_max_ = 1 μM, AUC = 4.2 μM·h). Compound **45** has shown activity on tumor progression in an isogenic K562 xenograft mice experiment (TGI around 46%), being patented for treatment of proliferative disorders, including myelodysplastic syndrome and leukemia (WO2023212181).

Overall, research on STK17 inhibitors remains very recent. Initial efforts were primarily directed toward STK17B inhibitors, with the first reports of selective STK17A inhibitors emerging only from 2021 onward. To date, the chemical space explored for both kinases remains highly limited, with only five distinct scaffolds described for STK17B inhibition and three for STK17A inhibition (**Table 3**). This narrow diversity highlights rather the novelty of the field than identified significant challenges associated with identifying drug-like and selective inhibitors.

Among STK17B inhibitors, the thieno[2,3-b] pyridine scaffold demonstrates promising two-digit nanomolar enzymatic potency; but exhibits limited selectivity over other DAPK kinase. In addition, physicochemical liabilities may constrain their use in biochemical or cellular studies. For example, the lead compound **16** displays a very low predicted solubility (cLogS = -6.5), likely due to its rigid structure and limited number of rotatable bonds. Experimental validation of solubility will be important to determine its suitability as a chemical probe. Future optimization could focus on the introduction of polar or solubilizing substituents, *e.g.* through further exploration of region II, which to date exhibits relatively flat structure-activity relationships.

The indirubin scaffold shows high inhibitory activity against STK17 kinases but suffers from poor selectivity. Moreover, structural features such as oxime moiety and overall molecular rigidity raise concerns regarding suitability in biological systems. These characteristics may negatively impact solubility and metabolic stability and could be associated with toxicity liabilities. Comprehensive evaluation of aqueous solubility, plasma stability, and potential oxime-related toxicity would therefore be essential before considering indirubin derivatives as viable chemical probes or therapeutic leads.

The benzofuran-3(2H)-one scaffold displays only modest submicromolar activity, with suboptimal selectivity even for the most advanced compound (**25**). Furthermore, the presence of a Michael acceptor motif, combined with phenol- or catechol-enriched substituents, raises concerns about chemical reactivity and stability under physiological conditions. Such features may lead to off-target reactivity or rapid degradation, limiting their applicability *in cellular* or *in vivo* studies.

Conversely, the 3-aminopyrazolylpyrimidine scaffold, and particularly its lead compound **27** (TRD-**35)**, displays more favorable physicochemical properties. Nevertheless, both potency and selectivity remain suboptimal and would require further optimization to support its development as a robust chemical probe.

Among the STK17B inhibitor series reported to date, the thieno[3,2-d] pyrimidine scaffold appears to be the most promising. Compounds from this series combine high two-digit nanomolar enzymatic potency with excellent selectivity over a broad kinase panel, including STK17A, and demonstrate sub micromolar cellular activity supported by direct target engagement using NanoBRET assays. In addition, the predicted physicochemical properties of the lead compound **29** are encouraging. The next steps toward validating this scaffold as a selective STK17B chemical probe will be the comprehensive characterization of its pharmacological profile *via in vitro* ADMET assays.

With respect to STK17A inhibitors, an initial aminopyrimidine scaffold has been described; however, the lead compound **31** still lacks sufficient potency and selectivity to qualify as a reliable chemical tool.

In contrast, the macrocyclic series — particularly the lead compound **40** (CK156) — represents a highly promising advancement. This scaffold exhibits two-digit nanomolar enzymatic potency that is largely preserved in cellular settings, with three-digit nanomolar activity observed *in cellulo*. Selectivity over other kinases also appears favorable, although direct comparisons across series remain challenging due to differences in selectivity metrics. Importantly, this series enabled the first co-crystal structures of STK17A, providing valuable structural insights into ligand binding within the catalytic pocket. It should be noted, however, that ligand-protein interactions observed for a given chemotype are not necessarily generalizable, and alternative scaffolds may engage STK17A through distinct binding modes. Additional structural and comparative studies across different chemotypes would therefore be highly valuable to the field.

Finally, the quinazoline-based scaffold represents a dual STK17A/B inhibitor series with a preference for STK17B. Although strong enzymatic inhibition has been reported for STK17B, residual activity against MYLK4 and STK17A limits the suitability of these compounds as selective chemical probes. Nonetheless, their demonstrated cellular activity and oral efficacy *in vivo*, particularly in acute myeloid leukemia models, support their further evaluation as preclinical oncology candidates. Notably, direct evidence of target engagement underlying the observed cellular and *in vivo* effects is still lacking and will be essential to establish the mechanistic basis of their pharmacological activity.

Overall, only a limited number of STK17 inhibitors have been reported, and substantial optimization is still required to develop truly ideal chemical probes. SGC-STK17B-1 (compound **29**) currently represents the most advanced candidate for studying STK17B inhibition, pending full pharmacological validation. However, there remains a clear unmet need for a highly selective STK17A probe. CK156 (compound **40**) is the most promising STK17A-directed compound to date, but further optimization is needed to achieve fully satisfactory potency, selectivity, and drug-like properties. An ideal STK17A chemical probe would combine single-digit nanomolar enzymatic potency with ≥50-fold selectivity over other kinases and possess physicochemical characteristics suitable for biological studies, including adequate solubility, favorable plasma and metabolic stability, and absence of obvious toxicophores. Crucially, robust evidence of target engagement *in cellulo* will be essential to directly link enzymatic or binding activity to observed cellular effects. This requirement is particularly important given that the biological consequences of selective STK17A inhibition remain largely unexplored. In this context, the use of selective chemical probes in genetically modified cell systems, such as STK17A knockout or siRNA-mediated knockdown cells, will be of highest interest for confirming known STK17A-specific functions, elucidating novel ones, and validating therapeutic hypothesis.

## Conclusion

STK17A, a member of the DAPK family within the CAMK superfamily, has emerged as a pivotal regulator at the intersection of apoptosis, cytoskeletal organization, immunity, and tumor biology. Its functions are highly context-dependent, acting either as a tumor suppressor or promoter depending on cellular environment and cancer type, and extending beyond oncology to influence autoimmune, infectious, cardiovascular, and neurodegenerative processes. Despite its physiological importance, progress in understanding STK17A has been hindered by the absence of the kinase in commonly used rodent models, as well as the reliance on *in vitro* overexpression or knockdown systems, which may not fully reflect physiological expression or localization. Mechanistic insights remain limited, with many reported associations lacking definitive evidence of kinase activity, substrate specificity, or pathway dependency.

STK17B, while structurally related, plays distinct biological roles, particularly in immune regulation, and transplant rejection. Dual STK17A/B inhibitors therefore carry the risk of producing confounding effects: inhibition of STK17B can lower T cell activation thresholds, enhance immune responsiveness, and disrupt homeostasis, potentially exacerbating autoimmunity or altering transplant outcomes. This highlights the necessity of selective chemical tools to interrogate STK17A independently and to avoid unintended immunological consequences.

Encouragingly, recent medicinal chemistry efforts have provided the first small-molecule inhibitors targeting STK17A and STK17B, including several dual inhibitors and a very limited number of selective compounds. The thieno[3,2-d]pyrimidine scaffold shows promise as a selective STK17B inhibitor, while the macrocyclic pyrazolopyrimidine compound **40** (CK156) has emerged as potential selective STK17A inhibitor. Nevertheless, the field still lacks optimized chemical probes that combine high potency, clear selectivity, and drug-like properties. Particularly, the development of new highly potent and selective STK17A inhibitors, sparing STK17B, is a key priority, as such compounds would enable precise functional studies, validate STK17A as a therapeutic target, and pave the way for novel interventions in cancer, autoimmune disorders, and neurodegenerative diseases. Beyond their use as chemical probes to dissect kinase-specific pathways, STK17A/B inhibitors, such as compound **45**, also hold potential as therapeutic leads for drug development, provided they display suitable pharmacological and safety profiles.

Complementarily, research should focus on generating physiological and tissue-specific STK17A models, including genetic loss- and gain-of-function systems, to delineate basal and stress-induced roles. Systematic identification of *bona fide* STK17A substrates and careful distinction between kinase-dependent and kinase-independent functions will be critical. Integrating these biological advances with the continued development of selective, well-characterized chemical probes will be essential for assessing STK17A's true translational potential.

In summary, STK17A represents both a promising and challenging kinase target. Fully realizing its therapeutic potential will require a synergistic approach that integrates structural biology, chemical biology, and translational studies, supported by potent, selective chemical tools. Such efforts have the potential to illuminate STK17A's multifaceted roles and unlock novel therapeutic opportunities across oncology, immunology, and neurodegeneration.

## Figures and Tables

**Figure 6:**
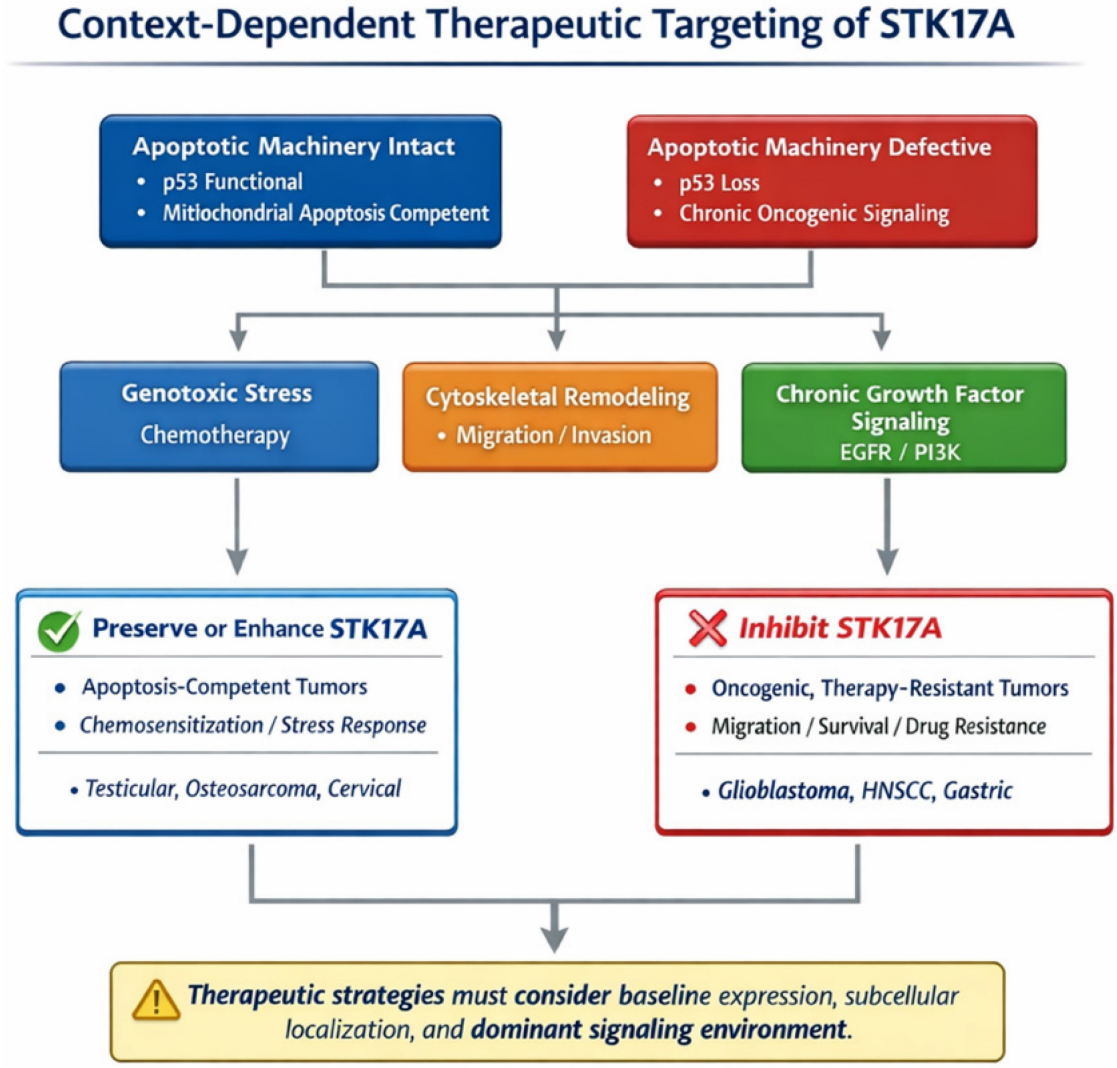
**Context-dependent therapeutic targeting of STK17A in cancer.** Decision framework scheme for context-dependent therapeutic targeting of STK17A in cancer. In apoptosis-competent tumors with intact p53 function and preserved mitochondrial apoptotic machinery, STK17A appears to act as an apoptosis amplifier and stress-response effector, supporting chemo sensitization under genotoxic stress. In contrast, in tumors characterized by defective apoptotic pathways, chronic oncogenic signaling, and cytoskeletal remodeling, STK17A may function as a non-oncogene dependency that promotes survival, migration, and therapy resistance. Accordingly, therapeutic strategies may favor preservation or enhancement of STK17A activity in apoptosis-competent contexts, whereas pharmacological inhibition may be advantageous in oncogenic, therapy-resistant settings. The scheme emphasizes that optimal targeting of STK17A requires consideration of baseline expression levels, subcellular localization, and the dominant signaling environment.

**Figure 7:**
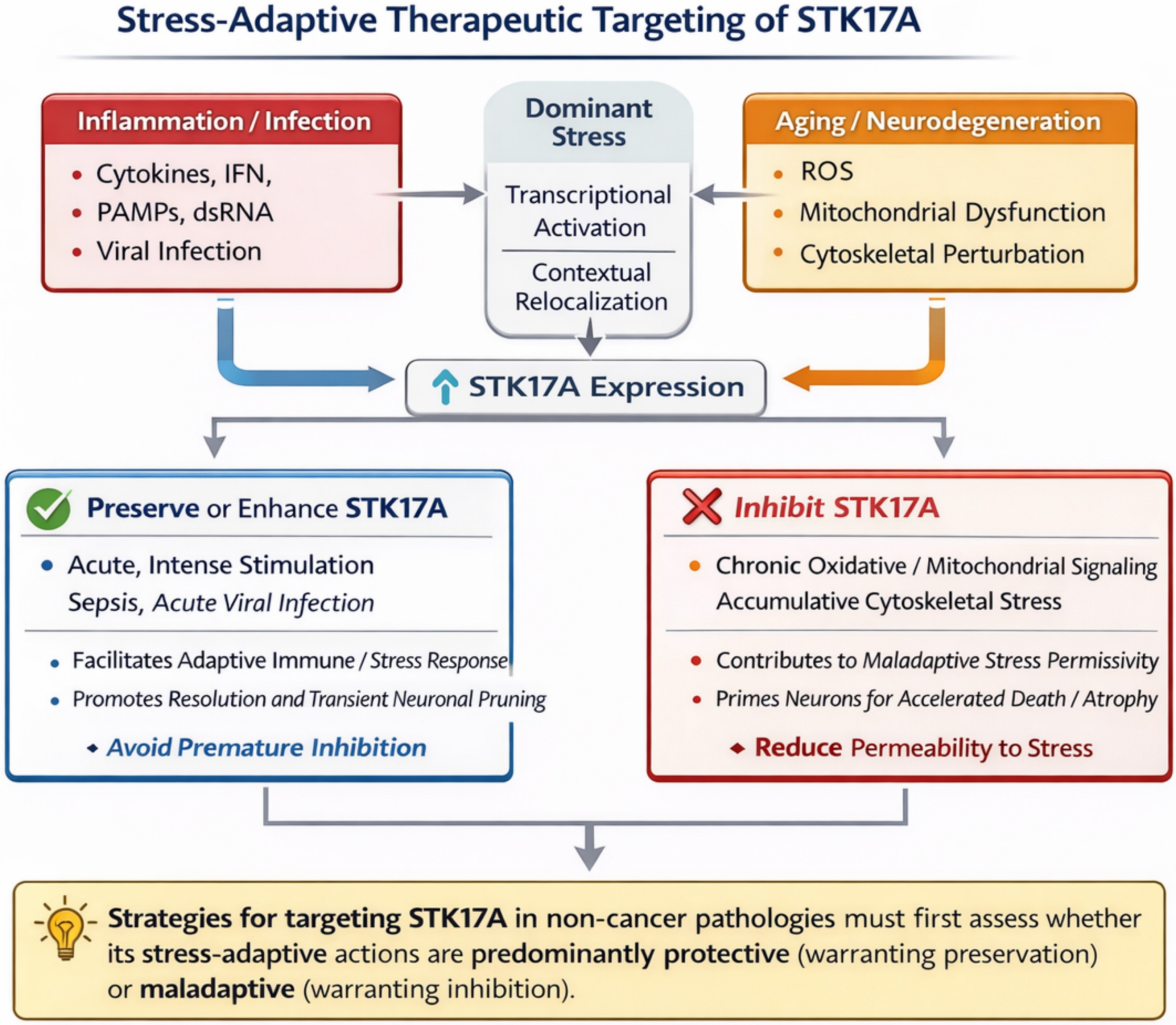
**Stress-adaptive therapeutic targeting of STK17A in non-cancer pathologies.** This schematic proposes a stress-adaptive framework for therapeutic modulation of STK17A in non-cancer pathologies. Diverse stressors, including inflammation or infection (cytokines, interferons, PAMPs, dsRNA) and aging- or neurodegeneration-associated stress (oxidative stress, mitochondrial dysfunction, cytoskeletal perturbation), converge on transcriptional induction and context-dependent relocalization of STK17A. In acute or intense stress settings, preservation or enhancement of STK17A activity may facilitate adaptive immune or stress responses and support resolution processes, suggesting that premature inhibition could be detrimental. Conversely, under chronic or cumulative stress conditions, sustained STK17A signaling may contribute to maladaptive stress permissiveness, neuronal vulnerability, or tissue degeneration, thereby providing a rationale for pharmacological inhibition.

**Table 1:**
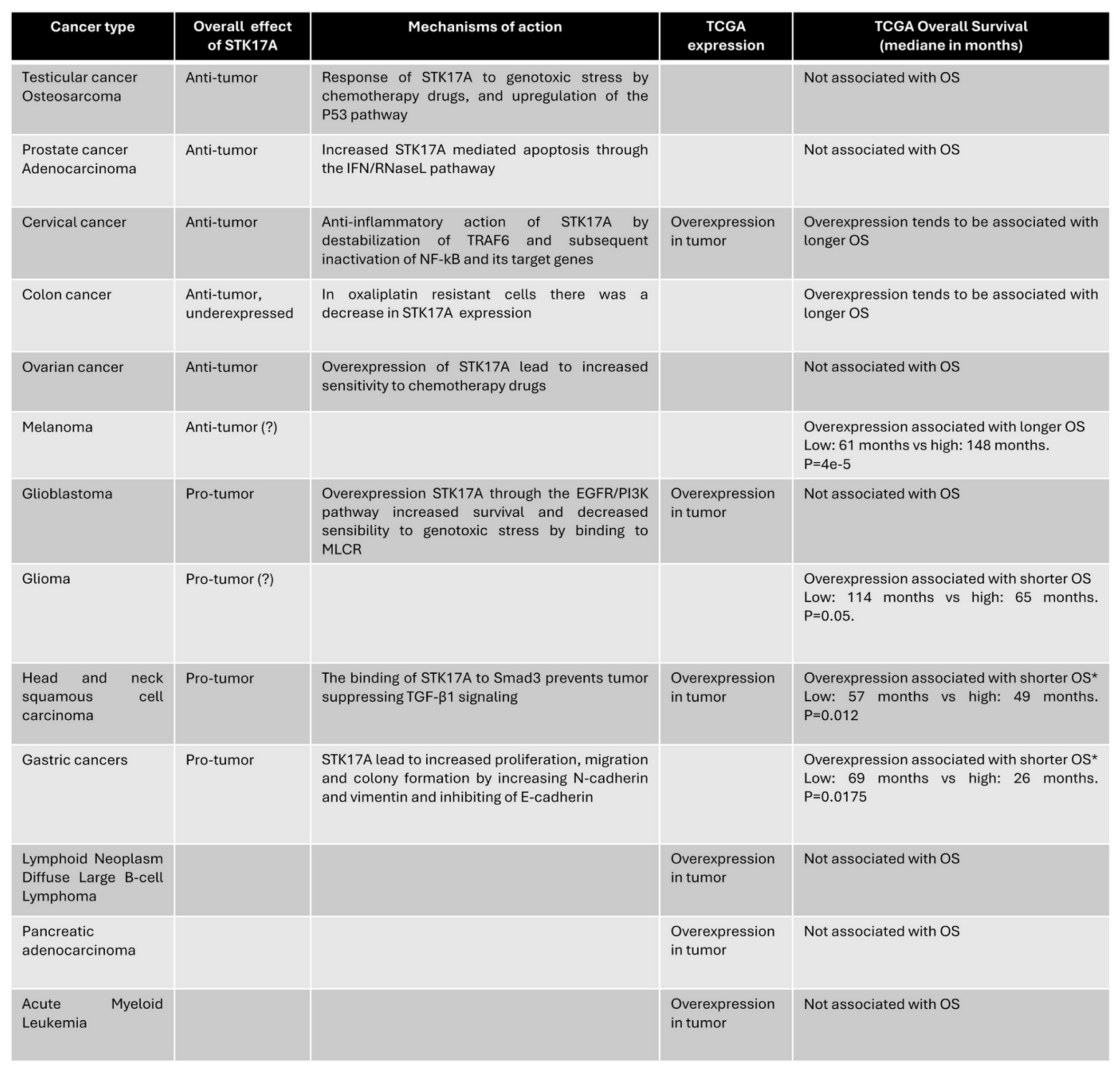
STK17A expression and effects in various cancer types

TCGA: The Cancer Genome Atlas; OS: overall survival.

**Table 2:** Comparative biological and therapeutic features of STK17A and STK17B

Feature	STK17A	STK17B
Genetic *in vivo* models	Absent (murine loss)	Robust knockout models
Dominant Biology	Stress/apoptosis, cytoskeleton	Immune tolerance, T-cell signaling
Cancer targeting logic	Context-dependent	Limited evidence
Autoimmune relevance	Indirect	Strong
Drug-development complexity	Higher (model limitation)	Lower

**Table 3:**
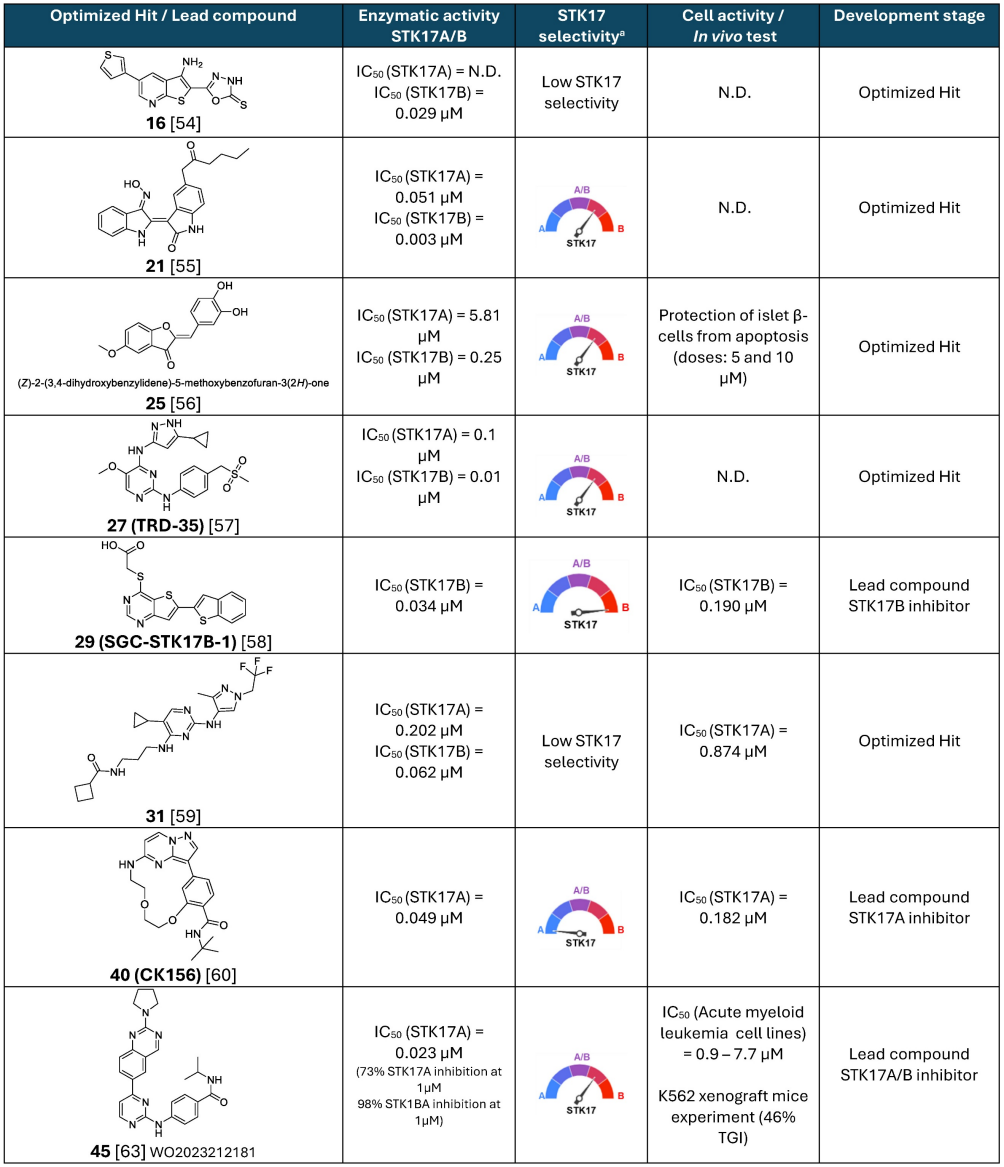
Summary of main small molecule STK17A and STK17B inhibitors.

N.D.: not determined. ^a^ Low STK17 selectivity: IC_50_ difference between STK17 and other kinases < 5-fold. STK17A/B selectivity: